# PQ-Neurolink: latency-aware post-quantum-ready secure communication for distributed brain–computer interfaces

**DOI:** 10.3389/fnsys.2026.1891041

**Published:** 2026-08-26

**Authors:** Tariq Qayyum, Asadullah Tariq, Abdelkader Nasreddine Belkacem

**Affiliations:** College of Computing and Artificial Intelligence, United Arab Emirates University, Al Ain, United Arab Emirates

**Keywords:** authenticated encryption, brain-computer interface, closed-loop latency, hybrid key exchange, ML-DSA, ML-KEM, post-quantum cryptography, secure transport

## Abstract

**Introduction:**

Distributed electroencephalography (EEG) brain-computer interface (BCI) systems increasingly transmit neural data and control messages between a head-worn device, a nearby hub, and cloud-assisted services. These links require integrity, authenticity, replay protection, and low latency, while migration to post-quantum cryptography can increase session-establishment cost.

**Methods:**

We present PQ-NeuroLink, a latency-aware post-quantum-ready communication framework that separates authenticated session establishment from the symmetric streaming fast path. The design supports classical X25519, post-quantum ML-KEM-768, and hybrid ML-KEM-768 plus X25519 key establishment, with pinned ML-DSA public keys for endpoint identity binding. The prototype was evaluated using EEG-derived traffic from public datasets over BLE-like and Wi-Fi-like link profiles, 1-hop and 2-hop topologies, symmetric key-update and public-key refresh events, active tampering scenarios, and MCU-informed resource and processing-energy projections.

**Results:**

Identity pinning and transcript binding prevented successful modeled session-splicing attempts. Post-quantum and hybrid modes primarily increased handshake bytes rather than steady-state streaming latency. In the most constrained BLE 1-hop summary condition, the hybrid M3 mode increased p95 latency by 0.45 ms relative to the unsecured M0 baseline while maintaining 99.0% packet delivery.

**Discussion:**

Post-quantum-ready authenticated session establishment can be integrated into distributed EEG communication prototypes when public-key operations are kept off the per-frame path. PQ-NeuroLink provides a reproducible communication-layer basis for secure next-generation BCI deployment studies requiring low-latency operation and long-horizon post-quantum protection.

## 1 Introduction

Brain–computer interfaces (BCIs) are steadily moving beyond laboratory demonstrations toward systems capable of supporting real actions, such as controlling a prosthetic or robot, selecting commands in assistive software, and providing rapid feedback to the user (Shanechi, 2019; Edelman et al., 2025). Among the available modalities, non-invasive electroencephalography (EEG) is especially attractive because it is relatively low-cost, portable, and already compatible with practical deployment pipelines in which a head-worn device streams neural signals to a nearby hub, such as a phone, tablet, or edge device, and in some cases onward to a cloud service for more computationally intensive processing (Schalk et al., 2004). At the same time, BCI systems are becoming increasingly closed-loop: the system senses brain activity, decodes user intent or state, and then immediately adapts what the user sees, hears, or feels, or adjusts stimulation in therapeutic settings (Zrenner et al., 2016; Soleimani et al., 2023; Topalovic et al., 2023). In such systems, timing is not a secondary implementation detail; it directly affects responsiveness, stability, usability, and, in some applications, safety.

While much of the BCI literature emphasizes signal quality, feature extraction, and decoding accuracy, practical deployment also depends critically on trustworthy communication. In a typical EEG-to-hub-to-cloud pipeline, the exchanged traffic may include raw or processed neural data, device state information, model outputs, and high-level control messages that trigger downstream actions. If an adversary can observe, modify, delay, or replay these messages, the closed loop can be disrupted, commands can be altered, and user feedback can become unreliable, which is especially concerning for assistive control and other safety-critical interactions (Bonaci et al., 2015). Prior work on BCI security and neurotechnology privacy has already highlighted risks across the BCI lifecycle, including malware exposure, data leakage, and integrity violations, and has argued for systematic security engineering rather than ad hoc protection mechanisms (Lopez Bernal et al., 2021). Nevertheless, most communication security deployed today still relies on classical public-key cryptography, such as RSA or elliptic-curve techniques, for authentication and key establishment.

This reliance on classical public-key mechanisms is becoming increasingly problematic because sufficiently large-scale quantum computers would break the assumptions underlying widely deployed schemes, thereby motivating the ongoing migration toward post-quantum cryptography (PQC). In response, NIST has standardized core post-quantum primitives, including ML-KEM for key establishment and ML-DSA for digital signatures (National Institute of Standards and Technology, 2024a,b). In many networked systems, the emerging practical pattern is to use PQC, often together with classical techniques in a hybrid migration mode, during session establishment, and then rely on efficient symmetric encryption for the sustained data path. For distributed EEG-based BCI communication, this suggests a natural architectural separation: an authenticated session setup between the head device and the hub, potentially extended to a cloud endpoint when needed, followed by a predictable low-overhead streaming phase in which EEG frames and control messages are protected using symmetric authenticated encryption. Rekeying can then be incorporated as a security-maintenance mechanism without placing expensive public-key operations on the continuous real-time path.

The central research gap is that BCI communication is inherently latency-sensitive, whereas PQC may increase handshake message size, computation time, memory pressure, and energy cost on constrained platforms. Even when PQC is confined to session establishment, reconnects, mobility, transient packet loss, and deliberate rekey policies can make the security layer visible to the user in the form of added setup delay, timing variance, or disrupted continuity. More fundamentally, BCI latency is not merely a decoder-side algorithmic quantity; it is an end-to-end systems property that includes sensing, buffering, wireless transmission, security processing, forwarding, and actuation. Prior work has shown that BCI latency can be measured and decomposed, but the literature still lacks a broadly adopted security-aware latency framework that connects representative BCI operating scenarios to explicit timing requirements and then evaluates whether contemporary PQC-capable communication designs can satisfy those constraints in practice (Wilson et al., 2010).

This paper addresses that gap by focusing on the head device-to-hub connection as the most exposed and timing-critical segment of the pipeline, while treating cloud connectivity as an optional extension for applications that require remote computation or storage. The study isolates the secure-channel layer through controlled EEG-derived traffic replay, enabling each protocol mode to be evaluated under identical packetization, loss, delay, reordering, topology, and attack assumptions. This design makes the latency and security effects attributable to the communication framework itself and provides a reproducible basis for subsequent platform-specific integration.

The paper establishes practical end-to-end latency and jitter targets for representative closed-loop EEG-based BCI use cases, grounding these targets in reported BCI timing measurements and relevant neuroscience constraints, and it formalizes a threat model covering integrity, authenticity, replay, impersonation, downgrade, compromised intermediary, cloud/API compromise, and future quantum-capable adversaries across the head–hub–cloud path. Building on these requirements, the paper presents and evaluates PQ-NeuroLink, a post-quantum-ready secure communication design with explicit symmetric key-update and public-key refresh policies intended to preserve security assurances within tight timing budgets. Public EEG datasets, including inner-speech resources, are used to generate traffic patterns, payload sizes, and update rates for a reproducible communication-layer evaluation. The main contributions of this paper are as follows:

- A BCI-specific secure-channel profile that separates authenticated session establishment from the low-latency streaming path, with explicit sequence, epoch, replay-window, and safe-mode behavior for EEG and feedback messages.
- A post-quantum-ready channel design with classical, post-quantum, and hybrid key-establishment modes, downgrade-resistant transcript binding, and clearly separated symmetric key updates vs. less frequent public-key refresh.
- A reproducible trace-driven evaluation methodology that uses public EEG datasets for traffic replay only, enabling controlled comparison of unsecured, classically secured, post-quantum, and hybrid communication modes under consistent link and topology assumptions.
- A systems-security analysis that combines latency percentiles, deadline miss rates, handshake wire size, amortized refresh cost, MCU-informed resource and processing-energy projection, statistical testing methodology, and a structured mapping between the threat model and the evidence provided by experiments or analytical arguments.

The novelty is therefore not the invention of a new cryptographic primitive or a replacement for TLS 1.3, QUIC, WireGuard, or IPsec. Rather, PQ-NeuroLink contributes a BCI-specific secure-channel profile and evaluation framework that binds PQC/hybrid session establishment, replay-aware packet acceptance, two-tier key maintenance, safety fallback, and EEG-derived latency budgets into a single communication-layer design. The remainder of the paper reviews related work on EEG–based BCI timing, neurotechnology security, secure healthcare/IoT networking, and post-quantum transport; defines the system model, latency and jitter metrics, and threat model; presents the PQ-NeuroLink architecture; describes the experimental setup; reports the performance, security, and analytical resource evaluation; and concludes with discussion and deployment considerations.

## 2 Related work

Electroencephalography-based brain-computer interfaces translate neural activity into device commands, typically through a pipeline that includes signal acquisition, preprocessing, feature extraction, decoding, and feedback to the user or actuator. Recent reviews emphasize that non-invasive EEG BCIs are moving beyond lab demonstrations into more realistic human-computer interaction, assistive control, and rehabilitation settings, but robustness, timing, and deployment constraints remain major barriers (Edelman et al., 2025; Hamad et al., 2024; Chen et al., 2020; Belkacem and Lakas, 2021). In parallel, new EEG decoders (often deep learning based) are enabling finer control granularity and more continuous interaction, including real-time robotic and prosthetic-like control loops (Skomrock et al., 2018). These advances make the BCI pipeline look increasingly like a safety-relevant cyber-physical system: brain-derived control signals are transmitted to devices, and devices return feedback (visual, haptic, or stimulation) that shapes subsequent brain activity, closing the loop (Thompson et al., 2014; Zrenner et al., 2016).

Latency and timing jitter are well-known determinants of BCI usability and learning. Standard performance measurement guidance in the BCI literature highlights that accuracy alone is not enough; timing, information transfer, and feedback dynamics directly affect end-to-end performance (Thompson et al., 2014). Several works provide procedures or empirical observations for measuring timing in BCI systems. (Wilson et al. 2010) proposed a procedure to measure component-wise and end-to-end latencies and jitter in typical online BCI configurations, and neural engineering studies have emphasized that stimulation, recording, and communication dynamics must be characterized explicitly rather than hidden inside aggregate performance metrics (Bossetti et al., 2004). More recent neuroimaging and EEG platforms emphasize that low and stable latency is necessary for real-time neurofeedback and brain-state dependent interaction, with modern open pipelines reporting tens of milliseconds end-to-end for EEG processing and feedback (Levitt et al., 2023). For specific EEG paradigms, feedback timing also affects performance; for example, P300 speller feedback frequency and timing influence ERP separation and task outcomes (Arvaneh et al., 2015). These timing studies motivate the engineering latency budgets used for the communication framework.

Security and privacy risks for BCIs have been discussed for more than a decade, with a key theme being that neural data and neural control channels carry sensitive information and can be misused. (Bonaci et al. 2015) framed BCI platforms as potential “app stores for the brain” and argued that neural signals and their derived commands require privacy and security protections across the full device-to-software lifecycle. (Lopez Bernal et al. 2021) provided a systematic security view of the BCI lifecycle and identified attack surfaces across acquisition, processing, communication, and application layers. Empirical work has demonstrated concrete risks: side-channel style EEG attacks can leak private information (such as PIN-related responses) from brain signals (Martinovic et al., 2012; Lange et al., 2018). Broader neurotechnology security discussions also connect these threats to safety and security applications and risks in operational environments (Brocal, 2023; Ienca and Haselager, 2016). While some recent work explores security at the wireless physical layer for brain-to-device communication using novel coding and metasurface-assisted approaches (Xiao et al., 2025), cryptographic end-to-end integrity and authentication across realistic BCI head-device to hub to cloud pipelines remains under-standardized, especially when considering future post-quantum adversaries.

Related healthcare and IoT security work reinforces the need to consider post-quantum migration together with constrained-device operation and network-layer attacks. A recent 6G smart-hospital architecture argues for multi-layer post-quantum protection in latency-critical medical infrastructures (Devaraj et al., 2026). In low-power IoT and RPL/6LoWPAN networks, routing-layer threats such as blackhole, lowered-rank, version-number, and flooding attacks are difficult to mitigate using cryptography alone, motivating simulation-driven and machine-learning-assisted detection approaches (Panda and Muthuraman, 2026; Panda and Supriya, 2023). Although these works do not target BCI telemetry, they are relevant because wearable EEG systems share several deployment constraints with medical IoT: intermittent wireless links, limited energy, constrained memory, and the need for safe behavior under routing or gateway compromise. Similarly, application-layer systems that combine phishing detection with cryptographic verification illustrate a broader pattern of combining data-driven detection with authenticity checks; this is cited only as a general cybersecurity example rather than as a BCI baseline (Reddy et al., 2025). Table 1 presents the comparison of timing, security and post-quantum coverage.

**Table 1:** Comparison of timing, security, and post-quantum coverage.

Work	EEG	Loop	Latency	Threat	Crypto	PQC	Rekey
(Wilson et al. 2010)	Y	Y	Y	N	N	N	N
Bossetti et al. (2004)	Y	Y	Y	N	N	N	N
(Thompson et al. 2014)	Y	Y	Y	N	N	N	N
(Skomrock et al. 2018)	Y	Y	Y	N	N	N	N
(Bonaci et al. 2015)	Y	Y	N	Y	Partial	N	N
(Lopez Bernal et al. 2021)	Y	Y	Partial	Y	Y	N	N
(Martinovic et al. 2012)	Y	N	N	Y	N	N	N
Lange et al. (2018)	Y	N	N	Y	N	N	N
(Xiao et al. 2025)	Y	Y	Partial	Y	Physical layer	N	N
**PQ-NeuroLink**	**Y**	**Y**	**Y**	**Y**	**Y**	**Y**	**Y**

Bold entries identify the proposed PQ-NeuroLink framework and the capabilities supported by the proposed framework.

Post-quantum cryptography (PQC) is becoming concrete through standardization and protocol integration. NIST has standardized key establishment and signatures designed to resist quantum adversaries, including ML-KEM for key encapsulation and ML-DSA and SLH-DSA for signatures (National Institute of Standards and Technology, 2024a,b,c). Internet security protocols are actively adopting hybrid and post-quantum transitions; TLS 1.3 defines a modern handshake and supports key updates for ongoing sessions (Rescorla, 2018), QUIC integrates TLS 1.3 and defines secure packet protection and update mechanisms (Thomson and Turner, 2021), and the IETF has standardized terminology for PQ/traditional hybrids (Internet Engineering Task Force, 2025). Implementations and benchmarks show that PQC can be practical on constrained devices, but handshake costs and message sizes can be significant compared to classical elliptic-curve key exchange, motivating careful engineering and hybrid approaches (Kannwischer et al., 2019; Stebila and Mosca, 2017; Schwabe et al., 2020).

For the embedded-device interpretation in Sections 3.3.4 and 4.7.1, we use the PQM4 framework and its current Cortex-M4 benchmark tables as cycle- and stack-level references rather than transferring the measured ARM64 execution times directly to a wearable power model. The PQM4 data are combined with representative active-current specifications for the 64-MHz nRF52840 and the 80-MHz STM32L4A6. This separates the measured hub-class results from a wearable-class projection in which execution time and CPU-processing energy are derived from the target clock frequency, supply voltage, and active current.

Table 2 clarifies how PQ-NeuroLink differs from existing general secure transports. The proposed framework should be interpreted as a BCI-specific secure-channel profile and evaluation methodology that can reuse ideas from TLS, QUIC, WireGuard, or IPsec, rather than as a claim that those mature protocols are inadequate in general. The distinction is that PQ-NeuroLink explicitly binds the protocol state variables needed by BCI telemetry—session identifier, epoch, message type, sequence number, timestamp policy, replay window, key-update cadence, and safe-mode transition—to EEG-derived latency budgets and trace-driven packetization.

**Table 2:** Comparison with general secure transports and PQC-enabled transport directions.

**System**	Identity and key establishment	Streaming protection	Key update/refresh	BCI-specific support	Role in this paper
TLS 1.3	Certificate/PSK; classical by default, PQ/hybrid groups emerging	Record protection over reliable stream	TLS Key update	No EEG traffic model or safe-mode semantics	Baseline and design reference
QUIC	TLS 1.3 handshake and transport parameters	Packet protection over UDP	QUIC key update	No explicit BCI deadline or replay policy	Baseline for datagram-like secure transport
WireGuard	Static public keys and Noise-style handshake	AEAD datagrams with counters	Timer/counter-based rekey	No PQC by default and no BCI safety interface	Low-overhead classical baseline
IPsec/IKEv2	PKI/PSK with IKE negotiation	Packet-level ESP protection	Security-association rekey	No EEG-specific latency profile	Network-layer baseline
PQ-TLS / KEMTLS directions	PQC or hybrid KEM/signature integration	TLS record or QUIC packet protection	Protocol-specific key update	Not specialized for EEG frame cadence	Cryptographic migration context
PQ-NeuroLink	Classical, ML-KEM, or hybrid establishment with pinned ML-DSA identity in the evaluated profile	AEAD with sid/epoch/type/sequence/timestamp associated data	Frequent symmetric key update and infrequent ML-KEM refresh	EEG-derived traffic, BCI deadlines, replay window, safe mode, and threat-evidence mapping	Proposed BCI communication-layer profile

A notable gap across these literatures is that BCI research has rigorous methods to measure and reason about latency and jitter (Wilson et al., 2010; Thompson et al., 2014), and security research has detailed threat surfaces and early demonstrations of neural data leakage and misuse (Bonaci et al., 2015; Lopez Bernal et al., 2021; Martinovic et al., 2012). PQC research provides standardized primitives and evolving protocol guidance (National Institute of Standards and Technology, 2024a; Rescorla, 2018; Internet Engineering Task Force, 2025). However, there is limited work that unifies (i) a closed-loop EEG–based BCI latency budget and acceptance framework, (ii) a realistic threat model including integrity of brain-to-device commands and device-to-user feedback, and (iii) an end-to-end post-quantum (or hybrid) security protocol design that is explicitly optimized to stay within those latency bounds.

## 3 Materials and methods

### 3.1 System model

**Figure 1:**
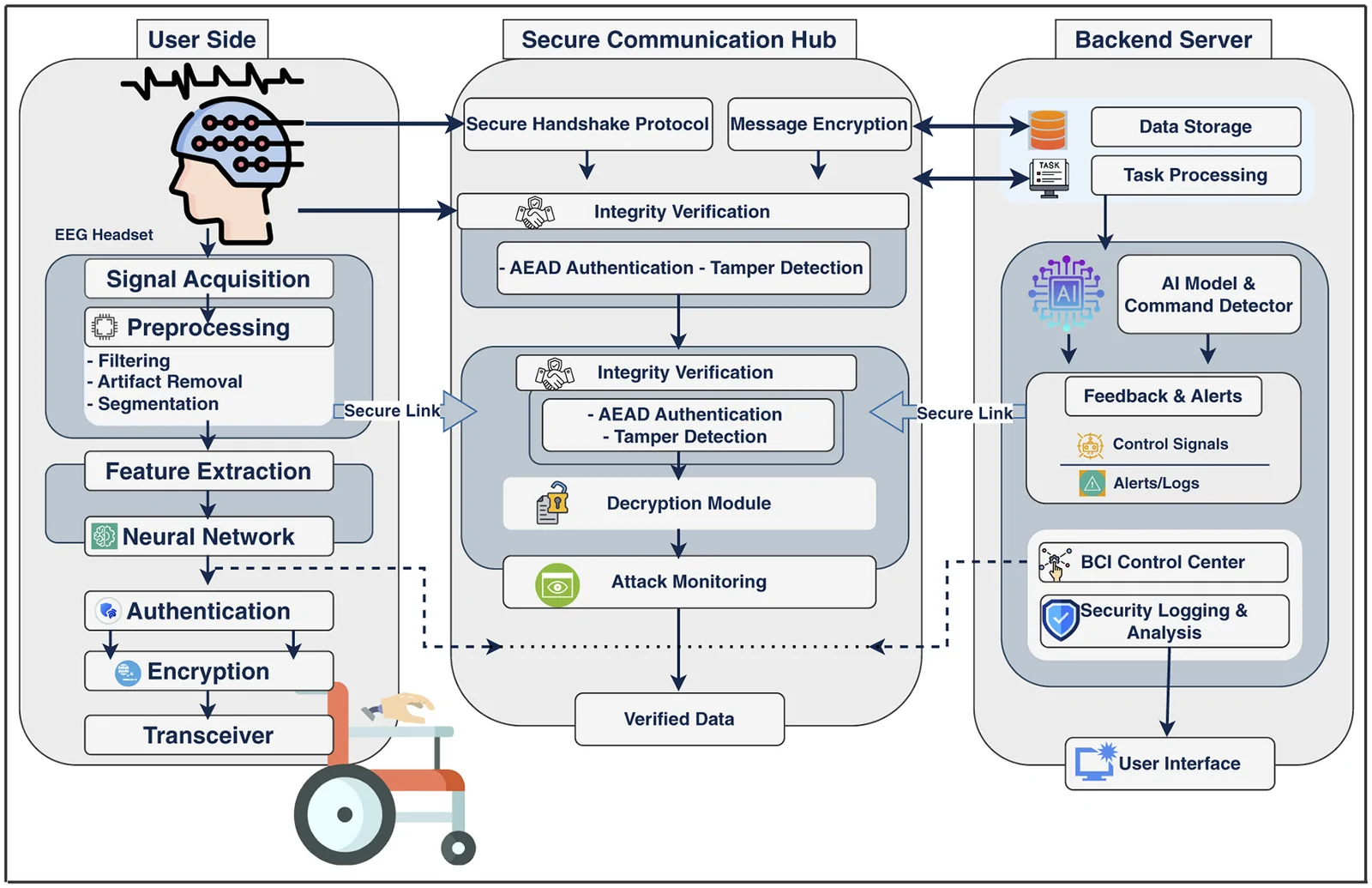
Secure closed-loop EEG-based BCI system architecture. Brain signal is acquired and optionally preprocessed on the user side, then authenticated and encrypted before transmission. A secure communication hub performs session establishment, message encryption and decryption, integrity verification through AEAD-based authentication and tamper detection, and attack monitoring. The backend server handles data storage and task processing, runs the AI model and command detector, and provides feedback or control signals, with security logging and alerts exposed through the BCI control center.

We model an EEG-based BCI as a closed-loop cyber-physical pipeline comprising three components, a head-worn device *D*, responsible for signal acquisition and optional lightweight preprocessing, a nearby hub *H*, which performs decoding and application logic, and optional cloud services *C*, used for computationally intensive inference, storage, personalization, or model updates. Communication is bidirectional. The uplink carries EEG frames, extracted features, decoder outputs, and control-relevant messages, while the downlink carries feedback, actuation acknowledgments, user-interface updates, haptic signals, or stimulation parameters. In this setting, the integrity and authenticity of command and feedback traffic are safety-critical, while confidentiality protects neural privacy and reduces long-horizon “harvest-now, decrypt-later” exposure (Martinovic et al., 2012). A list of notations used throughout this paper is provided in Table 3. Figure 1 illustrates the overall secure closed-loop architecture: EEG signals are acquired and optionally preprocessed on the user side, then authenticated and encrypted before transmission. A secure communication hub performs session establishment, message encryption and decryption, integrity verification through AEAD-based authentication and tamper detection, and attack monitoring. The backend server supports data storage and task processing, runs the AI model and command detector, and returns feedback or control signals, while security logging and alerting are coordinated through the BCI control center.

**Table 3:** List of notation and symbols.

Symbol	Meaning (units)
*f*_*s*_	EEG sampling rate (Hz)
*C*	Number of EEG channels
*T*_*f*_	Streaming frame duration / period (s)
*W*_*f*_	Samples per channel per frame, *W*_*f*_ = ⌊*f*_*s*_*T*_*f*_⌋
*W*	Decoder window duration (s)
*T*_*u*_/*f*_*u*_	Decoder update period / rate (s, Hz), *f*_*u*_ = 1/*T*_*u*_
*B*	Acquisition block duration (s)
*B*_τ_	Payload size for message type τ (bytes)
*B*_ *hdr* _	Protocol header bytes (bytes)
*B*_ *tag* _	AEAD tag bytes (bytes)
*B*_ *wire* _	On-wire bytes per protected packet (bytes)
$t_{i}^{\text{event}}$	Time of task-relevant neural event for update *i* (s)
$t_{i}^{\text{act}}$	Time at which the command is applied at the actuator/application for update *i* (s)
$t_{i}^{\text{per}}$	Time at which feedback is perceived or stimulation is delivered for update *i* (s)
$L_{\text{cmd}}^{\left(i\right)}$	Command latency for update *i* (s)
$L_{\text{fb}}^{\left(i\right)}$	Feedback latency for update *i* (s)
$L_{\text{loop}}^{\left(i\right)}$	Closed-loop latency for update *i* (s)
*J*	Jitter metric (s), representing latency or inter-arrival dispersion
*Q*_*p*_(·)	*p*-Quantile operator (e.g., *Q*_0.95_ denotes p95)
$L_{\max}^{\left(p\right)}$	Application tail-latency budget at quantile *p* (s)
$J_{\max}^{\left(p\right)}$	Application tail-jitter budget at quantile *p* (s)
*L*_AEAD_	Per-packet AEAD compute overhead (s)
*L*_replay_	Per-packet replay-check overhead (s)
*C*_*r*_	Transient cost of a rekey/refresh event (s)
ρ	Fraction of updates affected by rekey/refresh, ρ≪1
**1**_*r*_(*i*)	Indicator that rekey/refresh affects update or frame *i*
sid	Random session identifier
epoch	Traffic-key epoch counter
*s*_*i*_	Per-direction sequence number (monotone counter)
*R*	Replay-window size (packets)
Δ_*t*_	Optional timestamp acceptance bound (s)
*f*_osc_	Oscillation frequency for phase-locked loops (Hz)
*V*_DD_	Processor supply voltage used for the MCU projection (V)
*I*_act_	Active processor current under the selected workload (A)
*f*_clk_	Target microcontroller clock frequency (Hz)
*C*_op_	Benchmark cycle count for a cryptographic operation (cycles)
*P*_act_ = *V*_DD_*I*_act_	Active processor power used for device-specific energy projection (W)
*E*_op_	Estimated CPU-processing energy for one cryptographic or key-update operation (J)
*M*_peak_	Peak working memory of a cryptographic operation or protocol phase (bytes)
*N*_safe_	Consecutive missed or stale critical updates required to enter SafeMode
*N*_rec_	Consecutive valid critical updates required for recovery from SafeMode
*T*_TSS_	Time from the last valid critical update to application of the safe-state command (s)
$T_{\text{local}}^{\text{safe}}$	Local actuator or stimulation shutdown time after SafeMode is issued (s)

The architecture in Figure 1 serves as a reference communication architecture. In the evaluation, *D*, *H*, and *C* are instantiated as logical roles in the prototype so that protocol state binding, key maintenance, replay protection, and latency effects can be measured under controlled and repeatable conditions. This separation keeps the analysis focused on the secure-channel layer while remaining compatible with later integration into specific EEG headsets, hubs, and cloud services.

#### 3.1.1 Communication model and latency requirements

We next define per-update timestamps and latency variables, decompose the closed loop into measurable components, express requirements as tail constraints, and derive budgeting and feasibility relations that connect component-level tail behavior and rekey spikes to end-to-end closed-loop guarantees. This formulation is consistent with established BCI timing and performance-measurement methodology (Thompson et al., 2014).

We distinguish message types τ∈T in Equation 1:

$$\begin{array}{l} T=\left\{\mathrm{EEG},\mathrm{FEAT},\mathrm{DEC},\mathrm{CMD},\mathrm{FB},\mathrm{CTRL}\right\}. \end{array}$$ (1)

The head device forms streaming frames of duration *T*_*f*_ seconds, yielding *W*_*f*_ = ⌊*f*_*s*_*T*_*f*_⌋ samples per channel in Equation 2. If samples are transmitted using *b* bits/sample, the raw EEG payload size is:

$$\begin{array}{l} B_{\mathrm{EEG}}=C\cdot W_{f}\cdot \frac{b}{8}. \end{array}$$ (2)

Feature frames of dimension *d* with *b*_*f*_ bits/value have size in Equation 3:

$$\begin{array}{l} B_{\mathrm{FEAT}}=d\cdot \frac{b_{f}}{8}. \end{array}$$ (3)

A protected on-wire packet carrying payload *B*_τ_ bytes has approximate size:

$$\begin{array}{l} B_{\mathrm{wire}}\left(B_{\tau }\right)\approx B_{\mathrm{hdr}}+B_{\tau }+B_{\mathrm{tag}}+B_{\mathrm{link}}, \end{array}$$ (4)

*B*_*link*_ in Equation 4 captures link- and transport-layer overhead, such as BLE ATT framing or UDP/IP headers. If packets of type τ are sent every *T*_τ_ seconds, the corresponding application-level bitrate contribution in Equation 5 is:

$$\begin{array}{l} R_{\tau }\approx \frac{8\cdot B_{\mathrm{wire}}\left(B_{\tau }\right)}{T_{\tau }}\;\text{(bits/s)}. \end{array}$$ (5)

Let, $t_{i}^{\text{event}}$ in Equations 6–8 denote the time of a task-relevant neural event, or an equivalent reference time, for update *i*. Let, $t_{i}^{\text{act}}$ denote the time at which the corresponding command is applied at the actuator or application, and let, $t_{i}^{\text{per}}$ denote the time at which feedback is perceived or stimulation is delivered. We then define:

$$\begin{array}{ll} L_{\text{cmd}}^{\left(i\right)} & =t_{i}^{\text{act}}-t_{i}^{\text{event}}, \end{array}$$ (6)

$$\begin{array}{ll} L_{\text{fb}}^{\left(i\right)} & =t_{i}^{\text{per}}-t_{i}^{\text{act}}, \end{array}$$ (7)

$$\begin{array}{ll} L_{\text{loop}}^{\left(i\right)} & =t_{i}^{\text{per}}-t_{i}^{\text{event}}=L_{\text{cmd}}^{\left(i\right)}+L_{\text{fb}}^{\left(i\right)}. \end{array}$$ (8)

To support engineering analysis, we decompose the command path into nonnegative components:

$$\begin{array}{l} L_{\text{cmd}}^{\left(i\right)}=L_{\text{acq}}^{\left(i\right)}+L_{\text{buf}}^{\left(i\right)}+L_{\text{proc}}^{\left(i\right)}+L_{\text{net}}^{\left(i\right)}+L_{\text{sec}}^{\left(i\right)}+L_{\text{act}}^{\left(i\right)}. \end{array}$$ (9)

Here, acquisition and buffering capture sampling, windowing, block formation, and queueing; processing captures preprocessing and decoder inference; network captures medium access, retransmissions, and routing; security captures per-packet AEAD overhead together with episodic rekey or refresh effects; and actuation captures UI, haptic, or stimulation output. The decomposition in Equation 9 follows standard BCI latency measurement practice while making the security contribution explicit (Bossetti et al., 2004).

We report jitter in two complementary ways: as dispersion in end-to-end loop latency and as irregularity in update arrivals. Define tail-based latency jitter as:

$$\begin{array}{l} J_{L}^{\left(p\right)}≜Q_{p}\left(L_{\text{loop}}\right)-Q_{0.5}\left(L_{\text{loop}}\right), \end{array}$$ (10)

and inter-arrival jitter at the hub as follows. Let *a*_*i*_ denote the arrival time of update or frame *i* at the hub; then:

$$\begin{array}{l} \Delta a_{i}=a_{i}-a_{i-1},\;J_{A}^{\left(p\right)}≜Q_{p}\left(\mathrm{\Delta a}\right)-Q_{0.5}\left(\mathrm{\Delta a}\right). \end{array}$$ (11)

These two notions capture different operational concerns: phase-locked stimulation and neurofeedback emphasize inter-arrival regularity, whereas general closed-loop control places greater emphasis on tail end-to-end latency (Zrenner et al., 2016).

We express application requirements as quantile constraints, typically for *p*∈{0.95, 0.99}:

$$\begin{array}{l} Q_{p}\left(L_{\text{loop}}\right)\leq L_{\max}^{\left(p\right)},\;Q_{p}\left(J\right)\leq J_{\max}^{\left(p\right)}. \end{array}$$ (12)

This choice reflects the fact that closed-loop BCI usability is governed more by latency and jitter tails than by mean values (Thompson et al., 2014).

If the decoder uses a causal window of duration *W* and acquisition emits blocks of duration *B*, then the acquisition latency has the conservative lower bound:

$$\begin{array}{l} L_{\text{acq}}≳\frac{W}{2}+B. \end{array}$$ (13)

The $\frac{W}{2}$ term in Equation 13 reflects the use of window-centered evidence common in stable online feature extraction, while *B* accounts for block availability. This relation provides a necessary feasibility check: no optimization in the communication or security stack can reduce end-to-end latency below the lower bound imposed by windowing and acquisition structure.

For tail budgeting, let $L_{\text{cmd}}=\overset{K}{\underset{k=1}{\sum }}L_{k}$ denote the sum of *K* nonnegative components corresponding, for example, to acquisition, buffering, processing, networking, security, and actuation. Choose component budgets ℓ_*k*_≥0 and violation probabilities ε_*k*_ such that,

$$\begin{array}{l} \overset{K}{\underset{k=1}{\sum }}\ell_{k}=L_{\max}^{\left(p\right)}\;\text{and}\;\overset{K}{\underset{k=1}{\sum }}\varepsilon_{k}\leq 1-p, \end{array}$$ (14)

and enforce

$$\begin{array}{l} \mathrm{Pr}\left[L_{k}>\ell_{k}\right]\leq \varepsilon_{k}\;\forall k. \end{array}$$ (15)

By a union bound, $\mathrm{Pr}\left[L_{\text{cmd}}>L_{\max}^{\left(p\right)}\right]\leq 1-p$, and therefore $Q_{p}\left(L_{\text{cmd}}\right)\leq L_{\max}^{\left(p\right)}$. This turns qualitative “latency budgeting” into a concrete engineering procedure: measure the tail behavior of each component and allocate slack where it is most effective or least expensive.

##### 3.1.1.1 Phase error constraint for oscillatory closed-loop tasks.

For phase-locked stimulation and oscillatory coupling, loop delay induces a phase error as in Equation 16:

$$\begin{array}{l} \Delta \phi =2\pi f_{\text{osc}}L_{\text{loop}}, \end{array}$$ (16)

$$\begin{array}{l} Q_{p}\left(\Delta \phi \right)\leq \phi_{\max}^{\left(p\right)}\;\Leftrightarrow \;Q_{p}\left(L_{\text{loop}}\right)\leq \frac{\phi_{\max}^{\left(p\right)}}{2\pi f_{\text{osc}}}. \end{array}$$ (17)

This directly connects physiology-driven phase constraints to an explicit tail-latency budget (Zrenner et al., 2016).

#### 3.1.2 Security overhead as a fast path plus rare spikes

We model the security contribution to latency as the combination of a small per-packet overhead on the streaming fast path and rare transient costs caused by rekey or refresh events on the control plane. This abstraction makes it possible to state sufficient conditions under which security processing remains compatible with the same tail constraints imposed on the overall closed loop.

The per-update security delay is modeled as:

$$\begin{array}{l} L_{\text{sec}}^{\left(i\right)}=L_{\text{AEAD}}\left(\tau_{i},B_{\tau_{i}}\right)+L_{\text{replay}}\left(R\right)+1_{r}\left(i\right)C_{r}, \end{array}$$ (18)

where *L*_AEAD_ denotes the per-packet AEAD computation time, *L*_replay_(*R*) captures replay-protection bookkeeping as a function of replay-window size *R*, and *C*_*r*_ captures the transient work associated with a rekey or refresh event in Equation 18.

A latency-safe rekey policy must satisfy the same tail constraint as the overall loop:

$$\begin{array}{l} Q_{p}\left(L_{\text{loop}}\right)\leq L_{\max}^{\left(p\right)}\;\text{even when rekey/refresh events occur.} \end{array}$$ (19)

Let ρ denote the fraction of updates affected by rekey or refresh events in Equation 19, with ρ≪1 in the intended operating regime. A conservative sufficient condition is:

$$\begin{array}{l} Q_{p}\left(L_{\text{loop}}|1_{r}=0\right)+C_{r}\leq L_{\max}^{\left(p\right)}\;\text{and}\;\rho \leq 1-p, \end{array}$$ (20)

which guarantees that even if all spike-affected updates fall within the worst (1−*p*) fraction of the latency distribution, the *p*-quantile remains bounded in Equation 20. This formulation is intentionally conservative, but it is useful because it gives an implementation-independent way to reason about rare rekey transients without requiring strong distributional assumptions.

#### 3.1.3 Threat model

We next specify attacker capabilities and formalize the authenticity and freshness rule that the secure channel must enforce.

The protected assets include raw EEG samples and derived features, such as bandpower summaries, CSP features, and ERP epochs; decoded intents and device-control commands; feedback and control signals, including stimulation parameters where applicable; model artifacts and calibration state; and device identities together with cryptographic secrets. EEG traffic is sensitive because session data may contain health-relevant and identity-relevant information, and prior work has shown that even consumer EEG can, under some conditions, leak private attributes (Martinovic et al., 2012; Bonaci et al., 2015). For closed-loop safety, however, the dominant operational risk is malicious modification or replay of commands and feedback (Lopez Bernal et al., 2021).

We assume attackers may include (i) nearby wireless adversaries that eavesdrop, inject, replay, reorder, or delay packets on the head–hub link; (ii) malware that compromises the hub; (iii) compromise of cloud services or exposed APIs; and (iv) update or supply-chain attackers that target firmware, models, or downgrade paths (Lopez Bernal et al., 2021). Denial-of-service and jamming cannot be prevented by cryptography alone, but the system is required to fail safely under authentication failures, integrity violations, or timing anomalies.

The evaluation evidence is deliberately separated from the threat model. Packet injection, replay, delay, MITM/session-splicing, and downgrade manipulation are modeled directly at the communication layer. Compromised hubs, compromised cloud services, malicious updates, and supply-chain attacks are addressed by architectural controls—key isolation, endpoint pinning, signed updates, rollback protection, audit logging, least-privilege cloud interfaces, and safe-mode policy—but they are not experimentally validated in this trace-driven study. The discussion and security evidence matrix explicitly mark these items as architectural or future-work evidence rather than as completed attack experiments.

Let a command packet be *p*_*i*_ = (type, payload, *t*_*i*_, *s*_*i*_) with timestamp *t*_*i*_ and sequence number *s*_*i*_. The receiver applies the following acceptance rule:

$$\text{Accept}(p_{i})\;=\;[\text{Auth}(p_{i})=1]\;\wedge \;[s_{i}\in W(R)]\wedge \;[|t_{i}-t_{\text{hub}}|\leq \Delta_{t}].$$ (21)

Here, W(R) denotes a sliding replay window of size *R*, and Δ_*t*_ is an optional timestamp-acceptance bound. Sequence numbers remain mandatory, whereas timestamps may be omitted when clock synchronization is weak or unreliable.

Finally, we include a long-horizon adversary that records encrypted EEG sessions and attempts later decryption or authentication compromise using quantum-capable cryptanalysis. Accordingly, the design targets both active attacks in the present and post-quantum resilience for session establishment and long-lived trust (National Institute of Standards and Technology, 2024a,b,c; Internet Engineering Task Force, 2025). Table 4 presents a summary of the threat model for closed-loop EEG-based Q25 BCI communication, highlighting potential security threats and vulnerabilities across the head device, hub, and cloud components of the system.

**Table 4:** Threat model summary for closed-loop EEG–based BCI communication across head device, hub, and cloud.

**Attacker class**	Example capabilities	Primary security goals
Nearby wireless attacker	Eavesdrop, inject, replay, reorder, or delay traffic on the head–hub link	Integrity and anti-replay for CMD/FB; confidentiality of EEG in transit
Compromised hub	Access to decoded commands, application logic, and keys in memory	Minimize hub trust; safe-mode behavior; auditability
Compromised cloud/API	Access to stored EEG, inference outputs, and update channels	Signed updates; least privilege; optional end-to-end encryption
Update/supply-chain attacker	Malicious firmware or model update; downgrade attempt	Authenticated updates; rollback protection
Downgrade attacker	Force negotiation from M3/M2 to M1/M0 or remove PQ algorithms from the transcript	Signed transcript binding; mandatory mode policy; fail closed on unsupported downgrade
Future quantum-capable adversary	Record now and later attempt decryption or signature compromise	PQC/hybrid key establishment; PQ-safe signing

### 3.2 System architecture and quantum-safe secure channel

We now translate the system requirements defined in Section 3.1 into a secure channel between the head device *D* and the nearby hub *H*, with an optional extension to the hub–cloud segment when remote computation or storage is required. The design follows a post-quantum-ready pattern: use public-key mechanisms primarily during session establishment and identity binding, and then protect the streaming plane using symmetric authenticated encryption so that per-packet latency remains predictable and tail behavior remains stable. This separation is central to the architecture. It allows the protocol to provide strong integrity, replay protection, confidentiality, and post-quantum resilience without placing heavyweight public-key operations on the continuous real-time EEG and feedback path.

#### 3.2.1 Protocol requirements derived from the model

We next derive concrete protocol requirements from the timing and threat model in Section 3.1. The goal is not only to provide confidentiality, integrity, authenticity, and anti-replay protection, but to do so in a manner that respects the tail-latency and rekey constraints expressed in Equations 12, 19. In particular, the secure channel must preserve the timing discipline of the closed loop while ensuring that command and feedback traffic remain strongly protected against tampering, replay, impersonation, and session splicing.

Accordingly, the protocol is required to provide: (i) integrity and authenticity for all message classes, with stricter freshness guarantees for CMD and FB traffic; (ii) anti-replay protection using monotone sequence numbers and a bounded sliding window; (iii) confidentiality for EEG and derived data while in transit; (iv) forward secrecy and post-quantum resilience for session establishment and long-lived trust; (v) rekey behavior that does not stall or destabilize the real-time stream; and (vi) fail-safe behavior under authentication failure, replay detection, or severe timing anomalies.

To support comparative evaluation, we define four security modes, summarized in Table 5. M0 is an unsecured baseline used only for comparison. M1 represents a classical secure-channel baseline. M2 is a post-quantum-only mode in which session establishment is based on ML-KEM and endpoint identity is bound using pinned ML-DSA public keys. M3 is a hybrid mode in which a classical ECDHE secret and an ML-KEM secret are combined during session establishment, while identity is again bound using pinned ML-DSA keys in the evaluated design. In all secured modes, the streaming data plane uses AEAD with explicit sequence numbers and replay-window enforcement.

**Table 5:** Security modes used for evaluation and deployment.

Mode	Key establishment	Authentication	Streaming protection
M0	None	None	None
M1	Classical ECDHE	Classical signatures or PSK	AEAD, seq, and replay window
M2	ML-KEM	ML-DSA (pinned)	AEAD, seq, and replay window
M3	Hybrid ECDHE + ML-KEM	ML-DSA (pinned)	AEAD, seq, and replay window

These modes capture the deployment progression most relevant to practice: from no transport protection, to classical protection, to PQ-only establishment, and finally to a hybrid migration mode that retains classical and post-quantum contributions during the transition period. Because all secured modes share the same low-latency symmetric data plane, the main performance differences are expected to appear during session setup, refresh, and rare control-plane events rather than during steady-state packet delivery.

#### 3.2.2 Message protection, acceptance, and replay defense

We now define the packet format, AEAD protection rule, nonce discipline, and replay-window semantics that implement the acceptance rule in Equation 21. The design objective is to make the streaming path simple, deterministic, and easy to validate under strict timing requirements.

Each application message is represented as:

$$\begin{array}{l} m_{i}=\left(\text{sid},\text{epoch},\tau_{i},s_{i},t_{i},x_{i}\right), \end{array}$$ (22)

where sid is a random session identifier, epoch identifies the active traffic-key epoch, τ_*i*_ denotes the message type, *s*_*i*_ is a monotonically increasing per-direction sequence number, *t*_*i*_ is an optional timestamp, and *x*_*i*_ is the plaintext payload in Equation 22.

Each message is protected using AEAD in Equation 23:

$$\begin{array}{l} c_{i}\leftarrow \text{AEAD.Enc}\left(K_{\text{epoch}},n\left(\text{epoch},s_{i}\right),{\text{aad}}_{i},x_{i}\right), \end{array}$$ (23)

where the associated data contains all header fields that must be integrity-protected, at minimum sid, epoch, τ_*i*_, *s*_*i*_, and optionally *t*_*i*_. Nonce uniqueness is enforced by the pair (epoch, *s*_*i*_), with *s*_*i*_ strictly increasing for each direction and each traffic key. This keeps nonce generation deterministic and avoids the need for randomness on the fast path.

The receiver accepts a message only if decryption succeeds and the message is fresh:

$$\begin{array}{l} \text{Accept}\left(m_{i}\right)=\left[\text{AEAD.Dec}\neq ⊥\right]\wedge \left[s_{i}\in W\left(R\right)\right]\wedge \left[|t_{i}-t_{H}|\leq \Delta_{t}\right], \end{array}$$ (24)

where W(R) is a sliding replay window of size *R*, and the timestamp term is enforced only when timestamp validation is enabled. Let *s*_max_ denote the largest accepted sequence number in the current epoch. Then a newly received sequence number *s*_*i*_ is eligible if and only if:

$$\begin{array}{l} s_{i}>s_{\max}-R\;\text{and}\;s_{i}\notin \text{(already-received set in the window)}. \end{array}$$ (25)

This rule provides deterministic replay rejection together with bounded tolerance to packet reordering. In particular, packets that arrive slightly out of order can still be accepted as long as they fall within the permitted window and have not previously been processed. This is important for wireless settings in which reordering may occur transiently even when there is no active adversary.

The use of (sid, epoch, *ands*_*i*_) also prevents accidental state confusion across reconnects and refresh events. A packet protected under an older epoch cannot be replayed into a later epoch without failing AEAD verification or replay-window checks, and packets from one session are not accepted under another session identifier. This state binding is especially important in closed-loop systems because silent cross-session reuse could otherwise manifest as incorrect commands or feedback rather than as an obvious transport failure.

#### 3.2.3 Key establishment, key schedule, and rekey strategy

We next define the session-establishment procedure for M1, M2, and M3, together with the key schedule and the two-tier rekey strategy that keeps public-key work off the streaming fast path.

The protocol supports classical, post-quantum, and hybrid session establishment. In M1, the shared secret is derived from a classical ECDHE exchange. In M2, the session secret is derived from an ML-KEM shared secret (National Institute of Standards and Technology, 2024a). In M3, a classical ECDHE shared secret and an ML-KEM shared secret are combined in accordance with hybrid migration guidance (Internet Engineering Task Force, 2025). Endpoint authentication is performed using pinned public keys, specifically ML-DSA keys in the evaluated PQ and hybrid modes, so that session establishment is bound to stable endpoint identity and is resistant to impersonation and session-splicing attacks (National Institute of Standards and Technology, 2024b). This pinning model is appropriate for device–hub relationships in which endpoints are provisioned in advance and where certificate-chain processing would add unnecessary handshake complexity.

The design also makes downgrade resistance explicit. The negotiated mode, supported algorithm list, endpoint identities, ephemeral public values, ML-KEM ciphertext or classical ECDHE share, transcript hash, session identifier, role labels, and protocol version are included in the signed transcript and in the HKDF context string. A receiver configured to require M2 or M3 fails closed if an attacker removes PQC algorithms, substitutes a lower mode, replays an older negotiation, or changes the advertised key-update policy. This states the concrete channel-binding rule used by implementations and provides a direct basis for symbolic verification.

#### 3.2.4 Key provisioning, lifecycle, and revocation

Pinned ML-DSA public keys are embedded in a broader identity lifecycle model. During manufacturing or clinical enrollment, each headset or hub receives a device identity key, preferably generated in a secure element or trusted execution environment when available. During pairing, the hub stores the headset public-key fingerprint and a human-verifiable device identifier. Clinical or home deployments maintain an authorization registry mapping patients, headsets, hubs, and cloud services. Revocation is handled by removing the device fingerprint from the registry, distributing signed revocation lists or short-lived authorization tokens to hubs, and forcing re-enrollment after suspected compromise. Identity keys are rotated on a scheduled basis or after compromise, while traffic keys are updated through the symmetric and PQ refresh mechanisms described below. Audit logs record enrollment, revocation, failed authentication, downgrade attempts, firmware/model updates, and safe-mode transitions. This lifecycle model connects the pinned-key prototype to scalable device enrollment, revocation, and recovery procedures.

#### 3.2.5 Algorithm selection and cryptographic agility

The evaluated profile uses ML-KEM-768 because it provides a balanced category-3 key-establishment option, while ML-DSA-44 is used as a lower-overhead pinned identity signature in the prototype. The design is algorithm-agile: ciphersuite identifiers, transcript binding, and policy checks allow category-aligned or higher-assurance variants to be introduced without changing the streaming packet format. Deployments requiring category-3 authentication can select ML-DSA-65, while category-5 deployments can evaluate ML-KEM-1024 with ML-DSA-87 or SLH-DSA parameter sets. HQC is treated as a future backup KEM candidate because it was selected by NIST as a backup post-quantum encryption/key-establishment direction after the initial ML-KEM standardization process (National Institute of Standards and Technology, 2025). Table 6 summarizes the rationale for post-quantum parameter selection and deployment options.

**Table 6:** Post-quantum parameter-set rationale and deployment options.

Deployment profile	KEM	Signature	Rationale
Constrained prototype	ML-KEM-768	ML-DSA-44	Evaluated low-overhead profile; category-3 KEM with compact category-2 identity signature.
Category-aligned clinical profile	ML-KEM-768	ML-DSA-65	Recommended when category-3 authentication is required for long-lived device identity.
High-assurance profile	ML-KEM-1024	ML-DSA-87 or SLH-DSA	Higher security margin; requires new size, latency, and energy evaluation.
Future backup-diversity profile	HQC or hybrid KEM	ML-DSA/SLH-DSA	Code-based backup option for crypto agility and subsequent comparative evaluation.

In all secured modes, the protocol uses an HKDF-based schedule (Equations 26, 27) consistent with modern transport handshakes:

$$\begin{array}{l} \text{HS}\leftarrow \text{HKDF.Extract}\left({\text{salt}}_{0},Z\right), \end{array}$$ (26)

$$\begin{array}{l} \left(K_{\text{tx}},K_{\text{rx}},K_{\text{ctrl}}\right)\leftarrow \text{HKDF.Expand}\left(\text{HS},\text{info}\right), \end{array}$$ (27)

where *Z* is the session shared secret. Concretely, *Z* is the classical ECDHE secret in M1, the ML-KEM shared secret in M2, and a hybrid-combined secret in M3. The resulting keys are direction-specific and role-specific, allowing separation between the main traffic plane and the control or rekey plane.

Rekeying is split into two mechanisms: (i) frequent symmetric key updates, and (ii) less frequent public-key refresh. A symmetric update advances the traffic key with constant cost:

$$\begin{array}{l} K_{\text{epoch}+1}\leftarrow \text{HKDF.Expand}\left(K_{\text{epoch}},\text{bci-key-update}||\text{epoch}\right), \end{array}$$ (28)

triggered by elapsed time *T*_sym_, packet count *N*_sym_, or both. This operation is deliberately lightweight and is suitable for use during ongoing streaming without introducing noticeable disturbance.

A post-quantum refresh injects fresh entropy by running a new ML-KEM-based control-plane exchange:

$$\begin{array}{l} {\text{MS}}^{\prime }\leftarrow \text{HKDF.Extract}\left(\text{MS},Z^{\prime }\right), \end{array}$$ (29)

$$\begin{array}{l} K_{\text{epoch}+1}\leftarrow \text{HKDF.Expand}\left({\text{MS}}^{\prime },\text{info}\right), \end{array}$$ (30)

where *Z*′ is a fresh ML-KEM shared secret. The refresh cadence *T*_pqc_ is intentionally slower, for example on reconnect, on suspicion of compromise, or at coarse intervals such as tens of minutes, so that public-key processing remains off the per-frame fast path. This two-tier design matches the latency model of Section 3.1: symmetric updates contribute a small constant cost, whereas public-key refresh events are rare, explicit, and measurable control-plane spikes.

A symmetric key update is the inexpensive HKDF-only epoch transition in Equation 28. A public-key refresh is the less frequent ML-KEM-based control-plane exchange in Equations 29–30. A full re-handshake repeats authenticated session establishment after reconnect, policy failure, or suspected state compromise. The generic word rekey is used only as an umbrella term when referring to both symmetric key updates and public-key refreshes.

Algorithm 1 implements an offline, tail-budget-aware parameter search for the secure channel. Given the requirement tuple, including the target quantile *p*, measured baseline latency distributions for the non-security stages, link loss and reordering statistics, and an AEAD cost model, the algorithm enumerates candidate replay windows *R*, candidate symmetric update triggers (*T*_sym_, *N*_sym_), and candidate public-key refresh triggers *T*_pqc_. For each configuration, it estimates reordering-induced rejection risk for the selected replay window, predicts the security-delay contribution $L_{\text{sec}}^{\left(i\right)}$ under the corresponding rekey and refresh event model, and composes this with the measured or predicted distributions of acquisition, buffering, processing, networking, and actuation to obtain the resulting tail metrics *Q*_*p*_(*L*_loop_) and *Q*_*p*_(*J*). Only configurations that satisfy both tail constraints are retained. Among feasible configurations, the algorithm selects the one that maximizes a security-strength score, for example shorter exposure window, lower replay risk, and stronger refresh cadence, minus a performance penalty. The output is therefore a concrete parameter tuple (*R, T*_sym_, *N*_sym_, *andT*_pqc_) that is both timing-compliant and security-aware.

Algorithm 1 Tail-budget compliant selection of replay and rekey parameters. 
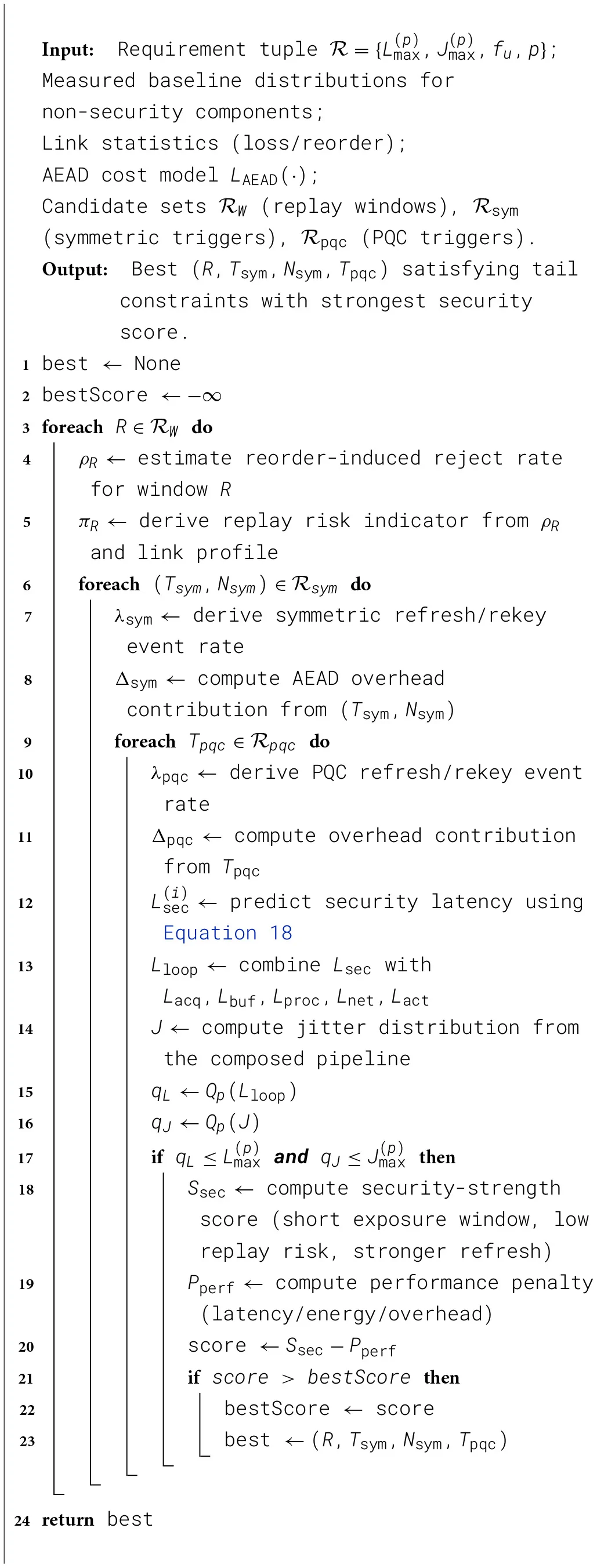

Algorithm 2 specifies the runtime behavior of the secure channel and separates the control-plane handshake from the low-latency streaming data plane. During the control-plane phase, the endpoints derive a shared secret *Z*, authenticate the peer, derive traffic and control keys, and initialize the session, replay, and safety-watchdog state. During streaming, each message is protected with AEAD and associated data binding (sid, epoch, τ, *s, t*). Cryptographic or protocol-state violations—including AEAD failure, replay, session mismatch, or epoch inconsistency—cause an immediate SafeMode transition. Timing-only anomalies use a hysteretic watchdog: one missed critical update raises a warning, two consecutive misses suppress new actuation and enter a degraded state, and the default threshold of *N*_safe_ = 3 consecutive missed or stale critical updates enters SafeMode. This distinction tolerates an isolated wireless loss while bounding continued operation under sustained delay or jamming.

Algorithm 2 Quantum-safe secure-channel runtime with bounded safe-state transition. 
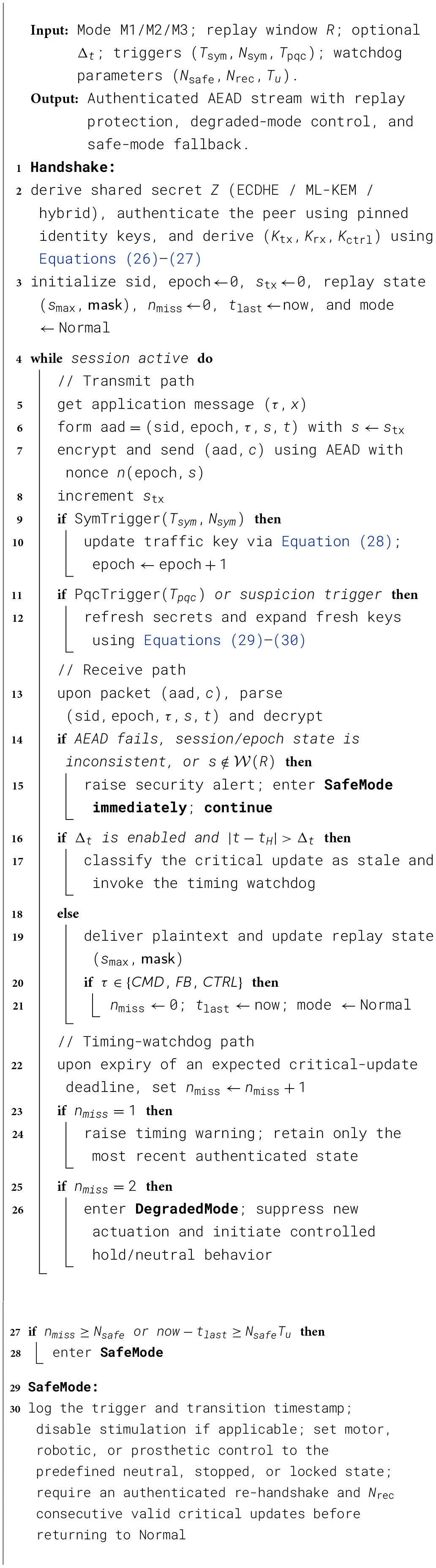

Let *n*_miss_ denote the number of consecutive missed or stale critical updates of type CMD, FB, or CTRL. The default communication policy uses *N*_safe_ = 3: the first miss raises a timing warning while retaining only the latest authenticated state; the second miss enters a degraded state that suppresses new actuation and initiates a controlled hold or neutral transition; and the third consecutive miss enters SafeMode, disables stimulation, and places motor, robotic, or prosthetic control in the predefined neutral, stopped, or locked state. The conservative time-to-safe-state bound is:

$$\begin{array}{l} T_{\text{TSS}}\leq N_{\text{safe}}T_{u}+T_{\text{local}}^{\text{safe}}, \end{array}$$ (31)

where *T*_*u*_ is the critical-update period and $T_{\text{local}}^{\text{safe}}$ is the local actuator or stimulation-controller shutdown time. In the evaluated per-frame feedback/acknowledgment configurations, *T*_*u*_ = *T*_*f*_∈{20, 40} ms, so the communication-watchdog component is bounded by 60 or 120 ms from the last valid critical update, before the local safe-state transition is added. A deployment satisfies a system-level safety budget $T_{\text{budget}}^{\text{safe}}$ when $N_{\text{safe}}T_{u}+T_{\text{local}}^{\text{safe}}\leq T_{\text{budget}}^{\text{safe}}$. Stimulation-bearing applications may select a stricter threshold, such as *N*_safe_ = 2, whereas non-actuating monitoring applications may tolerate a larger value after application-specific hazard analysis. Recovery requires authenticated session re-establishment followed by *N*_rec_ = 3 consecutive valid critical updates, preventing rapid oscillation between normal and safe states.

### 3.3 Experimental setup

#### 3.3.1 Datasets, traffic generation, and test conditions

This section describes the experimental topology, traffic generation process, protocol modes, network conditions, rekey schedules, attack tests, and measurement procedure used to evaluate latency, jitter, rekey impact, and attack resistance in a closed-loop EEG-BCI communication path. The latency and jitter terminology follows the system model and timing framework in Section 3.1, including the packetization and on-wire size definitions in Equations **??**–**??** and the tail-oriented jitter definition in Equations 11.

We evaluate an end-to-end pipeline with three logical nodes: a head device that streams EEG-derived traffic, a nearby hub that receives frames and produces feedback or acknowledgments, and an optional cloud endpoint for scenarios requiring remote computation. Experiments are run in a two-hop device–hub–cloud topology when cloud support is enabled and in a one-hop device–hub topology otherwise. The head–hub link is the primary focus because it is both the most exposed to nearby adversaries and the most timing-sensitive segment in typical wearable BCI deployments.

The evaluation intentionally isolates communication-layer behavior by replaying the same EEG-derived traffic through each security mode. This controlled setup makes protocol differences attributable to packetization, link model, key maintenance, and attack parameters rather than to vendor-specific acquisition hardware or radio firmware.

#### 3.3.2 Implementation, reproducibility, and measurement controls

Table 7 summarizes the implementation and measurement controls used to interpret the results. The table reports the software stack, protocol roles, replay design, network profile, repetitions, and timing interpretation so that the secure-channel evaluation can be reproduced consistently across protocol modes.

**Table 7:** Implementation and reproducibility details for the trace-driven evaluation.

Item	Reported configuration or interpretation
Evaluation type	Communication-layer replay using public EEG-derived traffic and identical protocol/link/topology conditions across all modes.
Logical roles	Head device *D*, hub *H*, and optional cloud *C* instantiated as software roles in 1-hop and 2-hop topologies.
Execution platform	A common ARM64 Linux test host is used for all reported protocol comparisons; cryptographic processing time is reported separately from link serialization and queueing delay.
Cryptographic stack	liboqs 0.15.0 for post-quantum primitives; Python cryptography 46.0.0 for classical baselines; AES-GCM for the symmetric fast path.
Benchmark repetitions	At least 400 measurements per reported data point; warm-up runs are discarded before timing; latency is summarized using median, p90, p95, p99, miss rate, and delivery rate.
Timing interpretation	Primitive and protocol-processing timings are separated from full communication latency; end-to-end results combine packetization, modeled link behavior, replay checks, AEAD, and key-maintenance events.
Link profiles	BLE-like profile: 1 Mbps nominal rate with constrained MTU and periodic transmission opportunities; Wi-Fi-like profile: 54 Mbps nominal rate with lower serialization delay and queueing jitter.
Loss and reordering	Low loss 0%–0.1%, moderate loss 0.5%, stressed loss 1%–3%; packet reordering is included to exercise replay-window behavior.
Randomization and reproducibility	Each protocol/link/topology/dataset condition uses fixed replay, delay, loss, and reordering seeds so that comparisons are paired across protocols.
Resource accounting	Measured ARM64 processing time and handshake bytes are reported separately from an MCU projection based on PQM4 cycle/stack data and representative 10–20 mW Cortex-M4 active-CPU profiles.

Public EEG datasets are replayed as realistic traffic sources and then packetized into frames that match practical acquisition settings. We use representative datasets spanning motor-imagery and inner-speech paradigms: (i) the PhysioNet EEG Motor Movement/Imagery dataset (EEGMMIDB), (ii) the BCI Competition IV datasets 2a and 2b, and (iii) the Thinking Out Loud inner-speech dataset. Table 8 summarizes the datasets and baseline acquisition characteristics used to drive the traffic generator. The role of these datasets is traffic generation: they provide realistic packetization rates, payload sizes, and temporal variability, while decoder benchmarking remains outside the communication-layer evaluation.

**Table 8:** Datasets used for realistic EEG traffic replay.

Dataset	Paradigm	*C*	*f*_*s*_
EEGMMIDB (PhysioNet, 2009)	Motor imagery	64	160
BCI Comp IV 2a (Brunner et al., 2008)	Motor imagery	22	250
BCI Comp IV 2b (Leeb et al., 2008)	Motor imagery	3	250
Thinking Out Loud (Nieto et al., 2022)	Inner speech	136	As recorded

For each dataset, samples are read in time order and framed into fixed-duration packets. The baseline operating points use *T*_*f*_∈{20ms, 40ms}, yielding *W*_*f*_ = ⌊*f*_*s*_*T*_*f*_⌋ samples per channel per frame. Raw-EEG frames use a fixed on-wire sample width, with 16-bit samples as the default setting, while feature frames represent reduced-bandwidth streaming configurations that avoid transmitting full raw EEG. Payload sizes follow the definitions introduced in the system model: raw EEG payload size is computed from Equation 2, feature payload size from Equation 3, and protected on-wire packet size from Equation 4. These two traffic types allow the evaluation to capture security overhead under both high-rate raw streaming and compressed feature streaming.

Security is evaluated across the modes defined in Table 5. The head–hub channel is instantiated in two deployment variants: (i) a datagram-style secure channel with a QUIC/TLS-inspired handshake and AEAD-protected application frames, and (ii) a QUIC-based prototype over Wi-Fi that reuses the standard QUIC security interface to TLS for key derivation and key updates (Thomson and Turner, 2021). The hub–cloud segment uses TLS 1.3 as a baseline (Rescorla, 2018), and, where available in the software stack, ML-KEM-enabled groups are used for post-quantum or hybrid negotiation (IETF TLS Working Group, 2026).

Wireless and network conditions are modeled using two link profiles. The first approximates BLE-like short-range streaming with constrained MTU and periodic transmission opportunities. The second approximates Wi-Fi-like streaming with higher throughput but variable queueing under contention. For each profile, a network emulator injects controlled delay variation, packet loss, and packet reordering. Results are reported across representative loss regimes: low loss (0%–0.1%), moderate loss (0.5%), and stressed loss (1%–3%). Because replay-window policy interacts with retransmissions and packet reordering, the experiments also record rejection attributable to replay-window enforcement in addition to any application-layer reassembly loss.

To exercise robustness beyond independent random loss, the evaluation framework also defines stress profiles with burst-loss episodes, intermittent connectivity requiring re-handshake, and multi-stream contention representing several EEG streams sharing a hub or access point. These stress profiles extend the validation envelope for replay-window sizing, key-refresh behavior, and safe-mode transitions under congested or intermittent wireless conditions.

Rekey behavior is evaluated using both time-based and packet-based triggers. Symmetric updates are performed frequently using the constant-cost update rule defined in the secure-channel section, while post-quantum refresh events are invoked less frequently to inject fresh entropy without requiring a full reconnect. Rekeying is parameterized by (*T*_sym_, *N*_sym_) for symmetric updates and *T*_pqc_ for post-quantum refresh. We sweep (*T*_sym_, *N*_sym_) from conservative to aggressive settings and measure the transient delay induced around rekey events. When extended key-update mechanisms beyond baseline TLS KeyUpdate are considered, they follow the intent of recent IETF efforts to strengthen update properties without incurring a full handshake restart (Tschofenig et al., 2026; Rosomakho et al., 2026).

Measurement follows established EEG–based BCI timing practice by timestamping pipeline stages and reporting full latency distributions rather than averages alone. For each frame index *i*, let $t_{i}^{\text{acq}}$ denote the timestamp at the end of acquisition for the last sample included in frame *i* at the head device, and let $t_{i}^{\text{fb}}$ denote the timestamp at which the corresponding feedback or acknowledgment becomes available again at the head. The measured end-to-end closed-loop sample-to-feedback latency is:

$$\begin{array}{l} L_{i}=t_{i}^{\text{fb}}-t_{i}^{\text{acq}}. \end{array}$$ (32)

For each condition, we report the median, p90, p95, and p99 of {*L*_*i*_}. Inter-arrival jitter at the hub follows the same tail-dispersion definition introduced in the system model; for convenience, we use:

$$\begin{array}{l} \Delta a_{i}=a_{i}-a_{i-1},\;J=Q_{0.95}\left(\mathrm{\Delta a}\right)-Q_{0.50}\left(\mathrm{\Delta a}\right), \end{array}$$ (33)

where *a*_*i*_ denotes the arrival time of frame *i* at the hub.

#### 3.3.3 Statistical analysis

For each protocol/link/topology/dataset condition, latency and jitter summaries are treated as paired repeated measurements because each protocol is evaluated on the same traffic source and link profile. We therefore use bootstrap confidence intervals and non-parametric paired tests rather than assuming normal latency distributions. Specifically, 95% confidence intervals for medians, p95, and p99 values are obtained using percentile bootstrap resampling of the per-frame latency vectors. Protocol comparisons are first screened using a Friedman test across the repeated protocol conditions, followed by paired Wilcoxon signed-rank tests for planned comparisons such as M3 vs. M2, M3 vs. WireGuard, and M3 vs. TLS/QUIC baselines. Holm–Bonferroni correction is applied to control family-wise error, and Cliff's delta or rank-biserial effect size is reported to distinguish statistical significance from practically negligible sub-millisecond differences.

Statistical interpretation is tied to paired per-frame vectors from the prototype. The reported tests therefore quantify how the security modes compare under identical replay and link assumptions, while platform-specific studies can reuse the same procedure to evaluate particular headset, hub, radio, or operating-system deployments.

Adversarial robustness is evaluated using four active communication-layer tests aligned with the threat model in Section 3.1: injection, replay, selective delay, and MITM/session splicing. For injection, replay, and MITM/session splicing, the primary security outcome is false acceptance of an unauthorized message or session. True rejection of malicious traffic and false rejection of benign traffic are reported separately. The delay test has different semantics: its outcome is the fraction of critical updates whose deadlines are violated, rather than successful cryptographic forgery. For delay and jamming-oriented conditions, the evaluation additionally records whether the watchdog reaches the expected warning, degraded, and safe states, the number of consecutive missed or stale critical updates at transition, and the analytical time-to-safe-state bound in Equation 31. With the default *N*_safe_ = 3 and the evaluated *T*_*u*_∈{20, 40} ms cadence, the communication-watchdog contribution is bounded by 60–120 ms from the last valid critical update, plus the platform-specific local shutdown time.

Cryptographic compute cost is considered separately from transport effects. For the protocol modes evaluated in this paper, handshake-processing time, verification cost, and rekey overhead are measured from the implementation or protocol stack used in the experiments whenever direct platform measurements are available. Published embedded benchmarks are used only to contextualize head-side feasibility and to compare the relative cost of PQC operations against symmetric AEAD, which supports the design decision to keep public-key operations off the per-frame fast path.

#### 3.3.4 MCU-informed resource and processing-energy accounting

The directly measured cryptographic timings in Section 4.1 characterize the common ARM64 evaluation host. A separate wearable-class projection is obtained from published Cortex-M4 cycle counts and stack measurements rather than by multiplying the ARM64 timing by a single assumed wattage. For an operation requiring *C*_op_ cycles on a target device *d* with clock frequency $f_{\text{clk}}^{\left(d\right)}$, the projected execution time is:

$$\begin{array}{l} t_{\text{op}}^{\left(d\right)}=\frac{C_{\text{op}}}{f_{\text{clk}}^{\left(d\right)}}. \end{array}$$ (34)

If the active processor current and supply voltage are $I_{\text{act}}^{\left(d\right)}$ and $V_{\text{DD}}^{\left(d\right)}$, respectively, then,

$$\begin{array}{l} E_{\text{op}}^{\left(d\right)}=V_{\text{DD}}^{\left(d\right)}I_{\text{act}}^{\left(d\right)}t_{\text{op}}^{\left(d\right)}=P_{\text{act}}^{\left(d\right)}\frac{C_{\text{op}}}{f_{\text{clk}}^{\left(d\right)}}. \end{array}$$ (35)

For a session with one authenticated handshake, $N_{\text{sym}}^{\left(s\right)}$ symmetric key updates, and $N_{\text{pqc}}^{\left(s\right)}$ public-key refreshes, the projected CPU-processing energy is:

$$\begin{array}{l} E_{\text{ctrl}}^{\left(d\right)}\approx E_{\text{HS}}^{\left(d\right)}+N_{\text{sym}}^{\left(s\right)}E_{\text{sym}}^{\left(d\right)}+N_{\text{pqc}}^{\left(s\right)}E_{\text{pqc}}^{\left(d\right)}. \end{array}$$ (36)

The wearable sensitivity analysis uses 10 mW and 20 mW active-CPU profiles. These values are grounded in representative Cortex-M4 specifications: the nRF52840 reports 3.3 mA at 64 MHz with its DC/DC regulator and 6.3 mA without that regulator for a CoreMark workload, corresponding to approximately 9.9 and 18.9 mW at 3.0 V, while the STM32L4A6 reports 37 μA/MHz at 3.3 V in SMPS run mode, or approximately 9.8 mW at 80 MHz (Nordic Semiconductor ASA, 2023; STMicroelectronics, 2022). ML-KEM-768 and ML-DSA-44 cycle and peak-stack values are taken from the current PQM4 benchmark tables (Kannwischer et al., 2019; PQM4 Contributors, 2026).

Equations 34–36 account for CPU processing only. Radio airtime can be parameterized separately as:

$$\begin{array}{l} E_{\text{radio}}\approx V_{\text{DD}}\left(I_{\text{tx}}t_{\text{tx}}+I_{\text{rx}}t_{\text{rx}}\right),\;t_{\text{tx}}≳\frac{8B_{\text{wire}}}{R_{\text{link}}}, \end{array}$$ (37)

with connection intervals, acknowledgments, retransmissions, and radio start-up time added for a specific BLE or Wi-Fi implementation. This separation is important because PQC increases handshake airtime, whereas the continuous EEG stream remains on the symmetric fast path.

## 4 Results

This section presents the communication-layer evaluation of PQ-NeuroLink across four publicly available EEG benchmark datasets, two radio link profiles (BLE-like at 1Mbps and Wi-Fi-like at 54Mbps), two network topologies (1-hop Device → Hub and 2-hop Device → Hub → Cloud), and nine security configurations ranging from no protection to hybrid ML-KEM-768/X25519 key establishment with pinned ML-DSA-44 identity binding. Post-quantum operations were implemented using liboqs 0.15.0, while classical baselines were implemented using Python cryptography 46.0.0. Unless otherwise stated, each reported data point is the median of at least 400 measurements, and error bars denote the interquartile range or the uncertainty interval available from the trace logs.

### 4.1 Post-quantum cryptographic benchmarks

The cryptographic values in this section isolate primitive and protocol-processing costs from the full communication path. Each primitive benchmark was run repeatedly after warm-up, with at least *N* = 400 measured repetitions. Optimized library implementations may use compiler optimizations and CPU instructions available on the ARM64 host; therefore the results are reported separately from packetization, link serialization, queueing, feedback rendering, and platform power management. To reduce ambiguity, the analysis separates (i) primitive compute time, (ii) handshake transcript size, and (iii) full communication latency.

**Figure 2:**
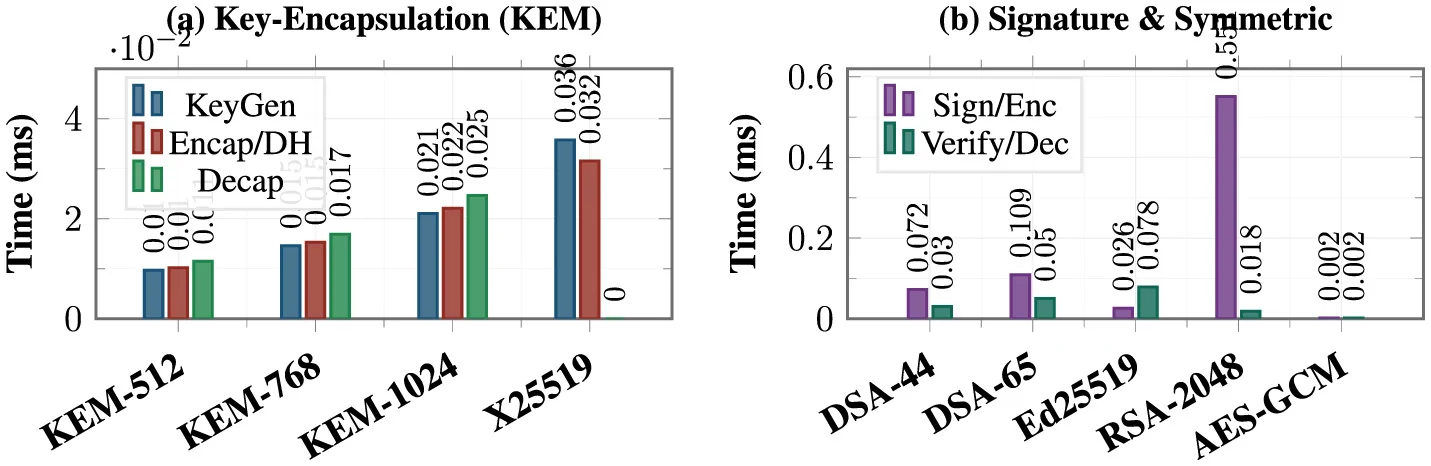
Measured execution times (mean, *N* = 400, ARM 64). **(a)** KEM operations: ML-KEM-512/768/1024 vs. X25519 ECDH. **(b)** Signature and symmetric operations: ML-DSA-44/65 vs. Ed25519, RSA-2048, and AES-256-GCM.

Figure 2 reports primitive-level cryptographic timings on the ARM64 software test environment. ML-KEM operations appear in the 0.010–0.025 ms range across the evaluated parameter sets, with ML-KEM-768 requiring 0.015 ms for encapsulation and 0.017 ms for decapsulation in the optimized library configuration. In the signature benchmarks, ML-DSA-44 signing and verification require 0.072 ms and 0.030 ms, respectively, while RSA-2048 signing reaches 0.551 ms in the same timing harness. The systems conclusion is that, in this prototype, public-key computation is separated from the per-frame path and is smaller than the link and packetization effects that dominate the communication latency results.

**Figure 3:**
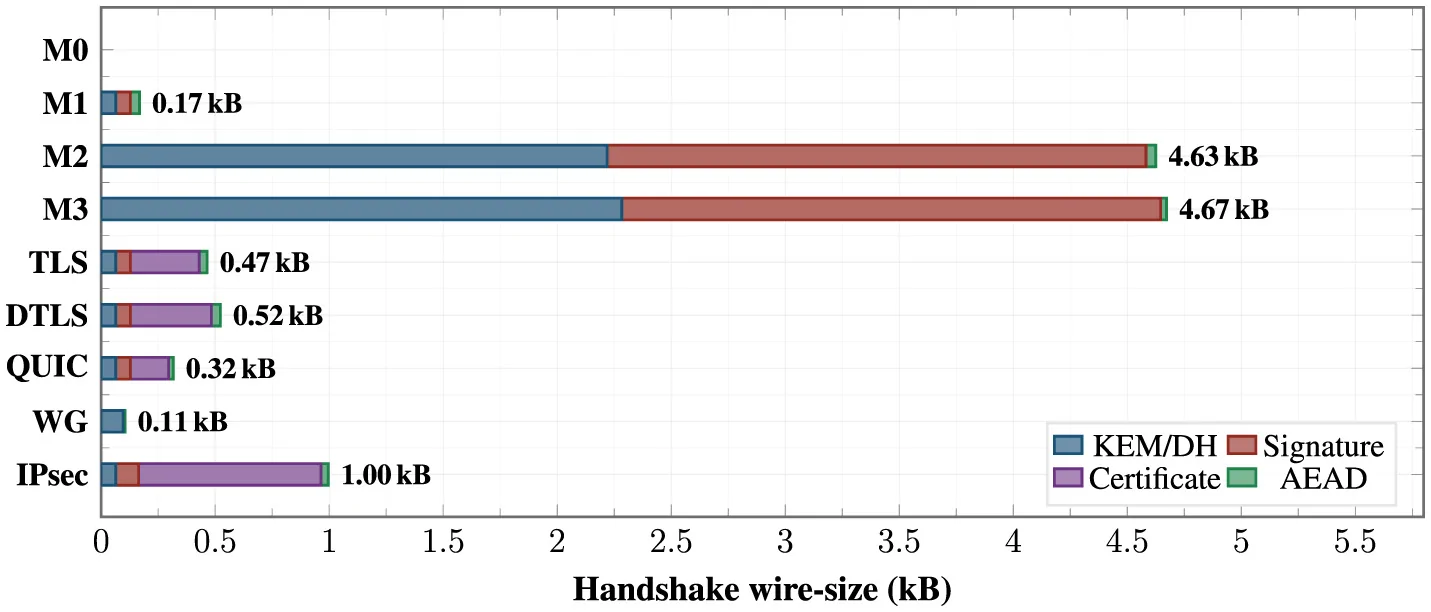
Measured handshake wire-size (kB) decomposed into KEM/DH key material, digital signature, certificate chain, and AEAD framing for all evaluated protocols. M2 and M3 have the largest handshake size, dominated primarily by the ML-DSA-44 signature contribution (approximately 2.4 kB each).

Figure 3 shows that the principal overhead of the post-quantum and hybrid modes lies in handshake transcript size rather than in the reported primitive-processing time. M2 and M3 require 4.63 kB and 4.67 kB, respectively, compared with 0.17 kB for M1, and this increase is dominated by ML-DSA-44 signatures and KEM key material. The corresponding measured cryptographic handshake-processing latency remains sub-millisecond for all evaluated PQ modes, with M2 at 0.149 ms and M3 at 0.182 ms. This separation explains why PQC primarily affects one-time setup and refresh events in the evaluated design, while the continuous EEG stream remains protected by the same symmetric fast path used across the secured modes.

### 4.2 End-to-end latency analysis

**Figure 4:**
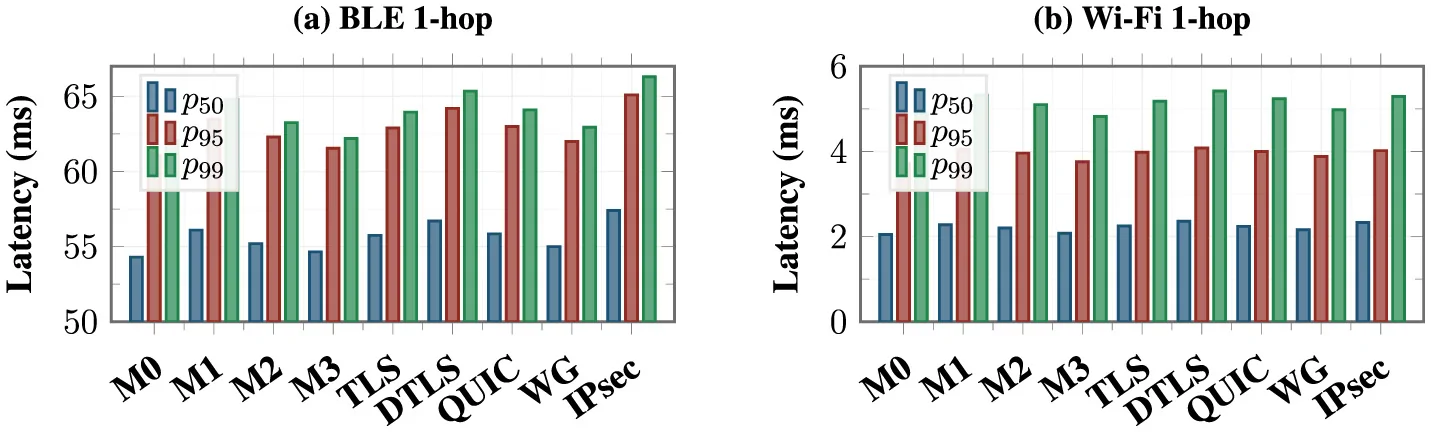
Measured end-to-end latency percentiles in the 1-hop setting across all datasets under **(a)** BLE and **(b)** Wi-Fi.

Figure 4 compares the measured end-to-end latency percentiles across all nine protocols in the 1-hop trace-driven setting. Under BLE, the latency spread across secured modes remains modest because link constraints and packet transmission time dominate the total delay. Within that setting, M3 records the lowest *p*_50_, *p*_95_, and *p*_99_ values among the secured schemes in this dataset/link configuration and remains close to the unsecured baseline M0, while DTLS and IPsec exhibit the largest tail values. Under Wi-Fi, where transmission delay is lower, the separation between protocols becomes clearer: M3 again gives the lowest percentile values among the secured modes in the evaluated traces and stays closest to M0 across all three percentiles.

The key interpretation is that when all secured modes share an AEAD-protected streaming fast path and public-key work is kept off the per-frame path, PQ or hybrid session establishment adds little steady-state latency in the tested conditions. Statistical comparisons are treated using the non-parametric procedure described in Section 3.3.3; sub-millisecond differences are emphasized as effect sizes and practical latency margins.

### 4.3 Statistical uncertainty and practical effect size

The latency results are interpreted using the bootstrap and paired non-parametric procedure defined in Section 3.3.3. In addition to reporting medians and tail percentiles, the manuscript distinguishes statistical uncertainty from practical relevance. For example, Table 9 shows that in the BLE 1-hop summary condition M3 differs from M0 by 0.45 ms at p95, while the full protocol spread is 4.00 ms. Such sub-millisecond differences may be statistically detectable with many frames but are not, by themselves, evidence of clinically meaningful improvement. Therefore, the main claim is restricted to feasibility: PQ/hybrid session establishment can be moved off the streaming fast path so that steady-state latency remains dominated by link, topology, and packetization effects under the tested traces.

**Table 9:** Summary of performance and security metrics (BLE, 1-Hop, all datasets).

Protocol	HS (ms)	HS(kB)	*p*_50_(ms	*p*_95_(ms)	DR(%)	**KEM cat**.
M0 (No Security)	0.000	0.00	54.30	61.10	99.69	–
M1 (X25519)	0.169	0.17	56.10	63.50	98.23	Classical
M2 (ML-KEM-768)	0.149	4.63	55.20	62.30	98.15	3
**M3 (Hybrid PQ)**	0.182	4.67	**54.65**	**61.55**	99.00	**3**
TLS 1.3	0.167	0.47	55.75	62.90	98.38	Classical
DTLS 1.3	0.167	0.52	56.70	64.20	97.99	Classical
QUIC	0.136	0.32	55.85	63.00	98.46	Classical
WireGuard	0.095	0.11	55.00	62.00	98.69	Classical
IPsec/IKEv2	0.634	1.00	57.40	65.10	98.53	Classical

HS, handshake; DR, packet delivery rate; KEM cat., ML-KEM key-establishment security category where applicable. ML-DSA-44 endpoint signatures are category 2. Overall *p*_95_ range across all protocols: 61.10–65.10 ms (spread: 4.00 ms). Bold text identifies the proposed M3 hybrid mode; bold p50 and p95 values are the lowest values among the secured schemes in the summarized BLE 1-hop condition. Bold formatting does not denote statistical significance.

The analysis also avoids claiming statistical significance for any condition where only aggregate plot values are available. Future artifact release should include the raw per-frame latency vectors, random seeds, and scripts for bootstrap confidence intervals, Friedman/Wilcoxon tests, Holm–Bonferroni correction, and effect-size computation.

### 4.4 Link type and network topology analysis

**Figure 5:**
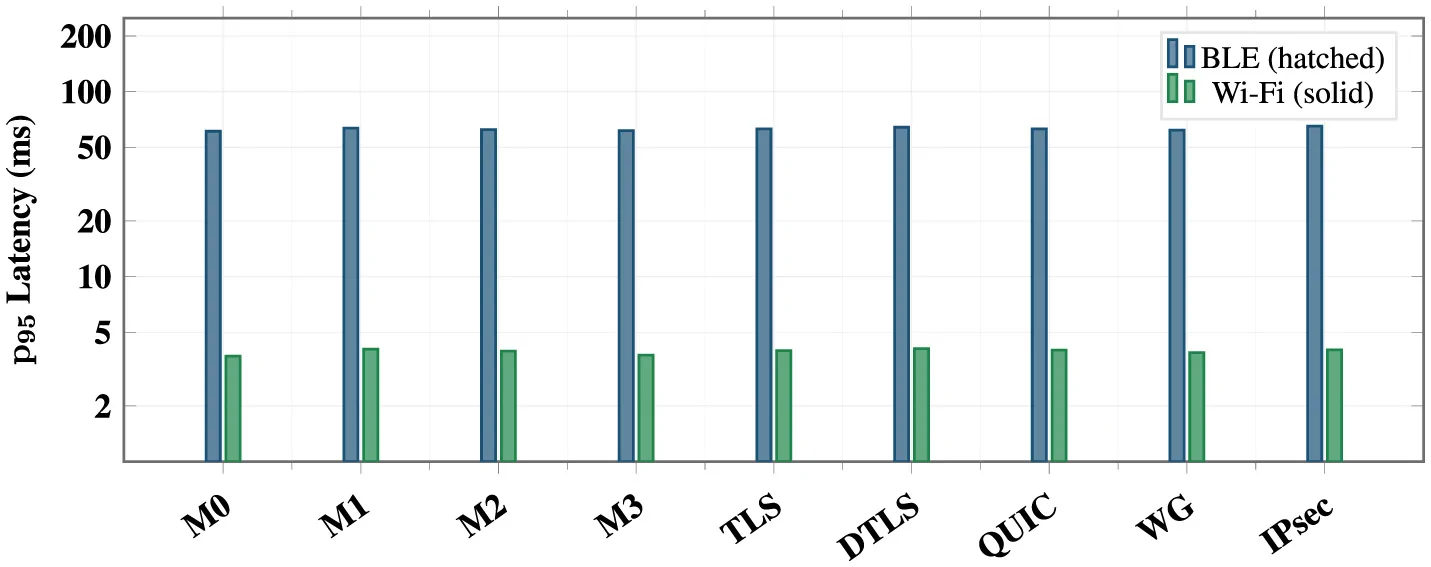
Measured *p*_95_ latency under BLE (hatched) and Wi-Fi (solid) for all nine protocols in the 1-hop setting. Link type dominates absolute latency, while M3 records the lowest *p*_95_ among the secured schemes in both link classes.

Figure 5 shows that link type is the dominant factor in end-to-end latency. For all protocols, BLE produces substantially higher *p*_95_ latency than Wi-Fi because lower link capacity and longer transmission time dominate the communication path. Within each link class, however, M3 records the lowest *p*_95_ among the secured schemes and remains closest to the unsecured baseline M0.

**Figure 6:**
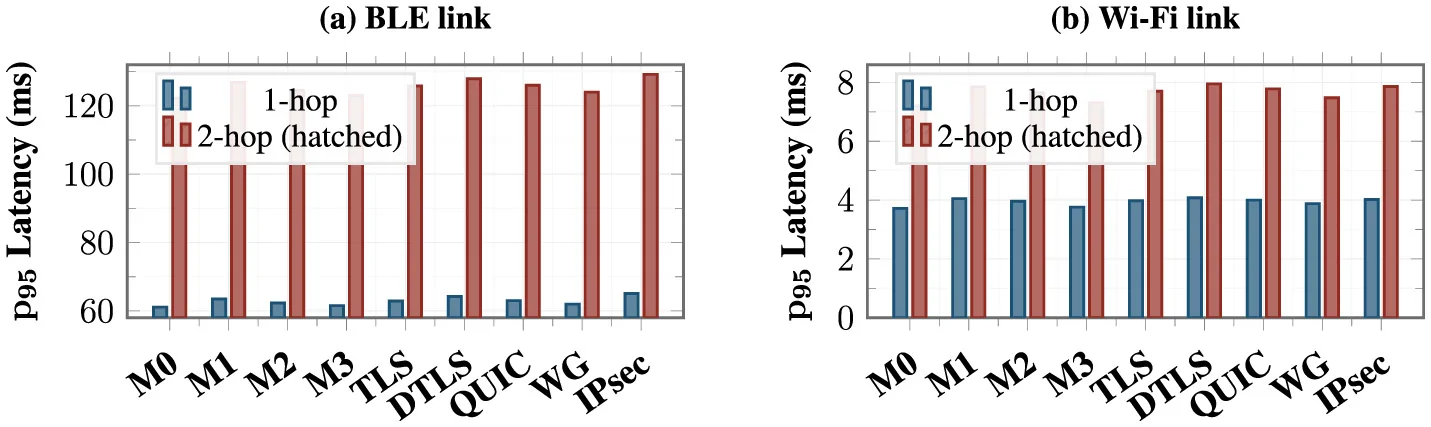
Measured *p*_95_ latency by protocol and topology for **(a)** BLE and **(b)** Wi-Fi links. Adding a second hop increases latency across all protocols, while M3 retains the lowest *p*_95_ among the secured schemes in both 1-hop and 2-hop settings.

Figure 6 shows that adding a second hop increases latency across all protocols on both BLE and Wi-Fi. The increase is larger on BLE, but the ordering across secured schemes remains consistent: M3 maintains the lowest *p*_95_, followed by WireGuard and M2, while DTLS and IPsec remain the highest-latency secured baselines. Overall, the measured results show that topology and link type dominate absolute latency, while the proposed hybrid mode remains the lowest-latency secured configuration across both deployment settings.

### 4.5 QoS deadline and miss-rate analysis

**Figure 7:**
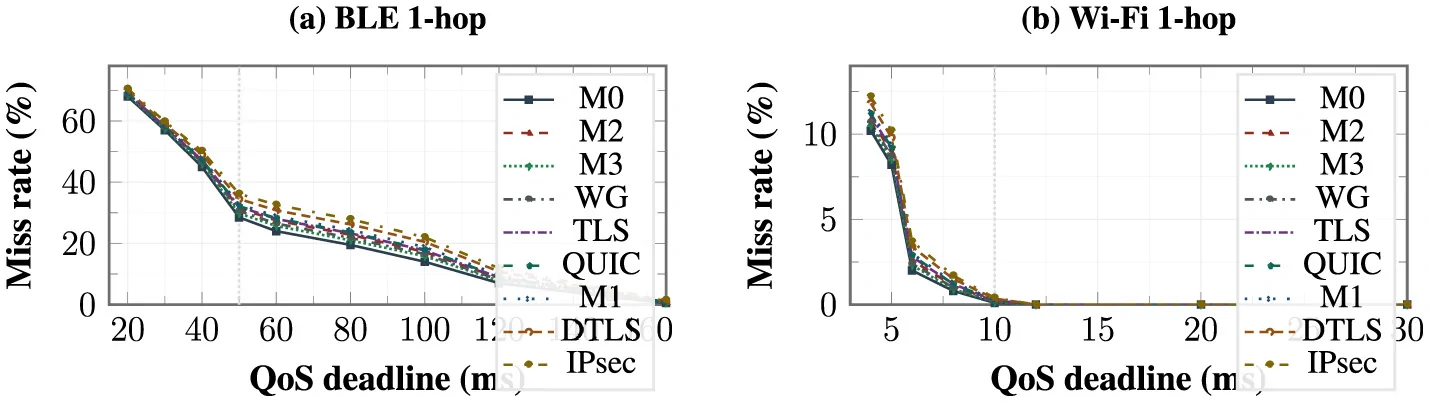
Measured packet miss-rate (%) versus QoS deadline in the 1-hop setting across all datasets for **(a)** BLE and **(b)** Wi-Fi. The curves separate most clearly in the transition region, where protocol overhead affects whether packets fall just above or below the deadline. M3 records the lowest miss-rate among the secured schemes on both BLE and Wi-Fi while remaining close to the unsecured baseline M0.

Figure 7 shows the measured packet miss-rate as the QoS deadline is varied. Under BLE, all protocols exhibit high miss-rates at very tight deadlines because the constrained link cannot deliver larger EEG frames fast enough. As the deadline relaxes, the curves separate most clearly in the 50–100 ms region, where protocol overhead determines whether packets fall just above or below the threshold. In this range, M3 records the lowest miss-rate among the secured schemes and remains close to the unsecured baseline M0, while DTLS and IPsec show the highest miss-rates.

Under Wi-Fi, the same pattern appears over a smaller deadline range because the link is faster and miss-rates drop rapidly once the deadline exceeds about 10–12 ms. Even in this tighter region, M3 again gives the lowest miss-rate among the secured protocols, followed closely by WireGuard and M2, while DTLS and IPsec remain the highest. Overall, the measured results show that the proposed hybrid mode provides the most favorable deadline performance among the secured schemes on both link types.

### 4.6 Security analysis

**Figure 8:**
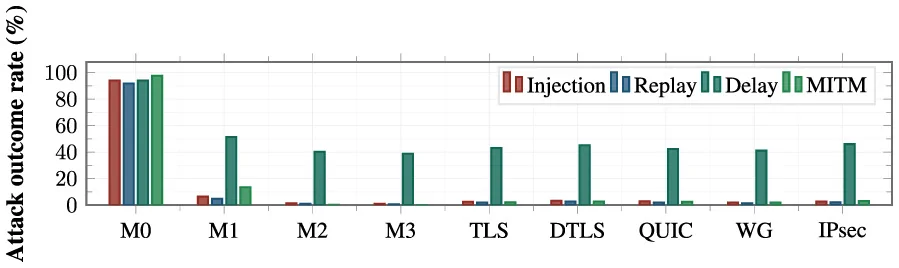
Communication-layer attack outcome rate (%) for Injection, Replay, Delay, and MITM/session splicing across all nine protocols. For Injection, Replay, and MITM/session splicing, the outcome denotes unauthorized acceptance or session compromise. For Delay, it denotes an induced critical-message deadline violation rather than cryptographic bypass. Under the default watchdog, three consecutive missed or stale critical updates trigger stimulation disablement or motor-control locking.

Figure 8 reports communication-layer attack outcome rates for Injection, Replay, Delay, and MITM/session splicing. For Injection, Replay, and MITM/session splicing, the outcome denotes unauthorized acceptance or session compromise; lower values therefore indicate stronger cryptographic protection. For Delay, the plotted outcome denotes an induced critical-message deadline violation, not a bypass of AEAD or endpoint authentication. M0 remains highly vulnerable, while M3 records the lowest outcome rates for the three cryptographic attack classes in the trace-driven tests. The comparatively high Delay bars show that authentication alone cannot preserve availability under sustained scheduling interference or jamming. Under the default watchdog, however, timing disruption cannot permit unbounded continuation of actuation: three consecutive missed or stale critical updates cause SafeMode, giving a communication-watchdog bound of 60–120 ms for the evaluated 20–40 ms update periods, plus $T_{\text{local}}^{\text{safe}}$.

**Figure 9:**
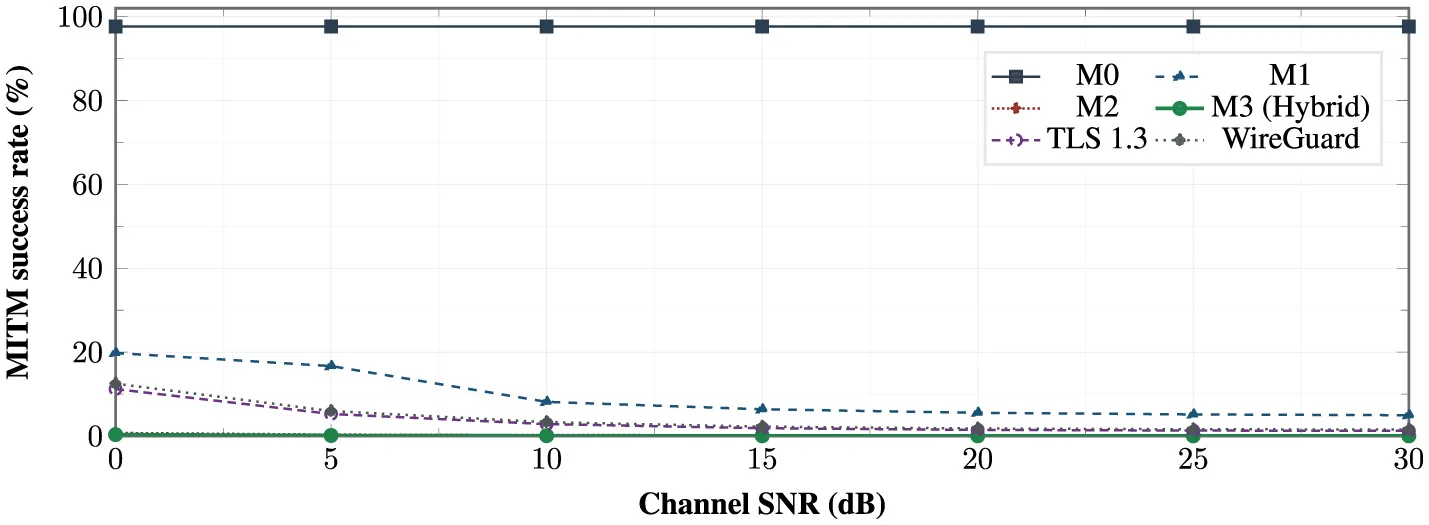
MITM success rate (%) vs. channel SNR. The hybrid mode M3 maintains the lowest success rate across the full SNR range, while M0 remains highly vulnerable.

Figure 9 shows the modeled MITM/session-splicing success rate as a function of channel SNR. The SNR axis is used as a link-quality stress variable in the emulator. Across the full modeled SNR range, M3 maintains the lowest success rate because the hybrid transcript and pinned ML-DSA identity binding make unauthenticated session substitution fail closed. This empirical result complements the transcript-binding rule and provides a concrete target for formal protocol verification.

**Figure 10:**
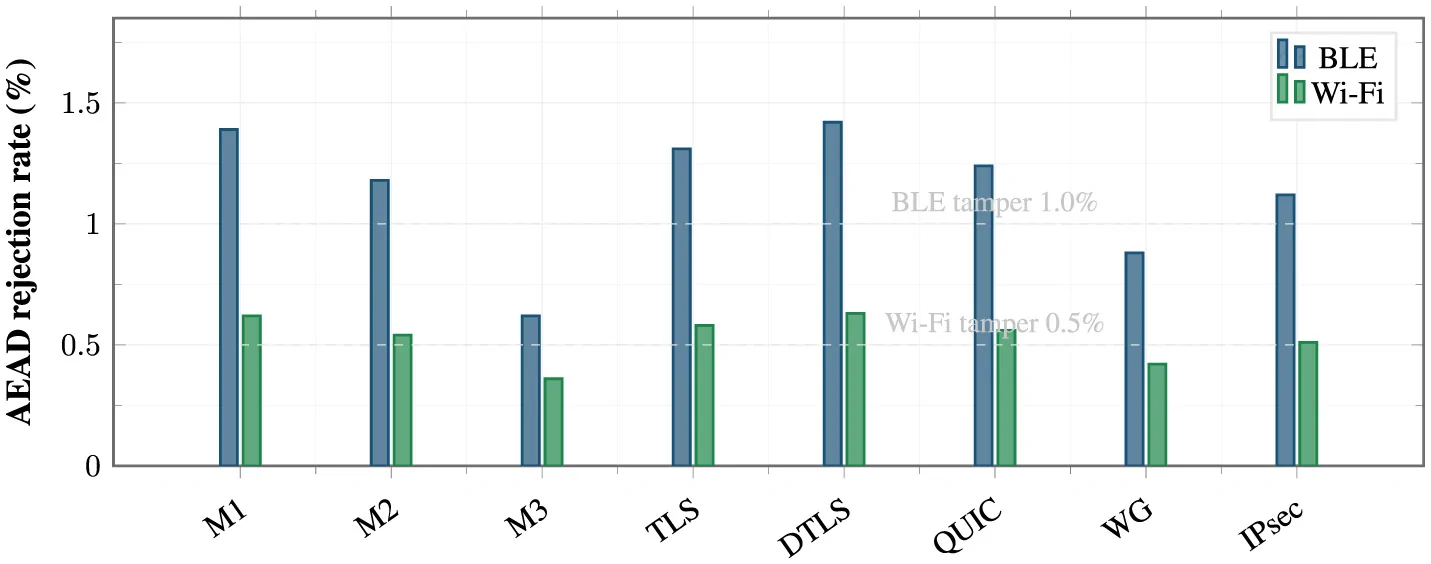
Measured AEAD authentication rejection rate (%) under BLE (hatched) and Wi-Fi (solid). M0 has no AEAD and is therefore omitted. M3 records the lowest rejection rate in both link types.

Figure 10 is interpreted as an auxiliary integrity and link-stress indicator. The primary security metric is false acceptance of malicious traffic; AEAD rejection distinguishes tamper detection and benign packet loss under the same modeled link conditions. The lower M3 rejection rate in the plotted conditions therefore indicates fewer rejected packets under the same modeled tamper profile, while the attack-success figures report the main security outcome.

#### 4.6.1 Structured security evidence

Table 10 maps each threat class to the corresponding design control and evidence type. The mapping clarifies which properties are exercised by the active communication tests and which are provided through architectural controls such as key lifecycle management, audit, signed updates, and safe-mode policy.

**Table 10:** Security claims, evidence type, and deployment considerations.

**Threat**	Design control	Evidence in this paper	Deployment consideration
Injection/modification	AEAD over payload and associated data including sid, epoch, type, and sequence	Modeled attack experiment	Radio-firmware adversary tests can instantiate the same packet-level checks on a target platform
Replay	Monotone per-direction sequence, replay window, epoch binding, optional timestamp bound	Modeled attack experiment and rule-based argument	Clock-drift and retransmission parameters are platform-specific and configure Δ_*t*_ and *R*
MITM/session splicing	Pinned identity keys, signed transcript, hybrid secret binding	Modeled attack experiment and protocol argument	Symbolic verification can further strengthen the protocol argument
Downgrade	Mode and algorithm list in signed transcript and HKDF context; fail-closed policy	Design argument; downgrade test specified	Interoperable implementation tests and symbolic analysis provide the next validation layer
Delay, intelligent jamming, or denial of service	Timestamp freshness, two-stage watchdog, degraded mode, *N*_safe_ = 3 consecutive-miss threshold, and bounded safe-state transition	Modeled delay experiment and analytical *T*_TSS_ bound	For 20–40 ms critical-update periods, the default communication-watchdog bound is 60–120 ms plus local shutdown time; stimulation profiles may select a stricter threshold
Compromised hub	Least privilege, key isolation, audit, revocation, safe mode	Architectural discussion	Strengthened by secure storage, process isolation, and audit on deployed hubs
Compromised cloud/API	Hub termination policy, signed updates, optional end-to-end protection, audit logs	Architectural discussion	Strengthened by cloud access control, audit, and optional end-to-end protection
Supply-chain/update attack	Signed firmware/model updates, rollback protection, revocation	Architectural discussion	Integrated through secure boot, signed updates, and rollback protection in product deployments
Future quantum adversary	ML-KEM/hybrid key establishment and ML-DSA identity in PQ modes	Cryptographic assumption and standards-based design	Parameter agility supports category-aligned and future standardized choices

### 4.7 Session-level overhead and delivery reliability

**Figure 11:**
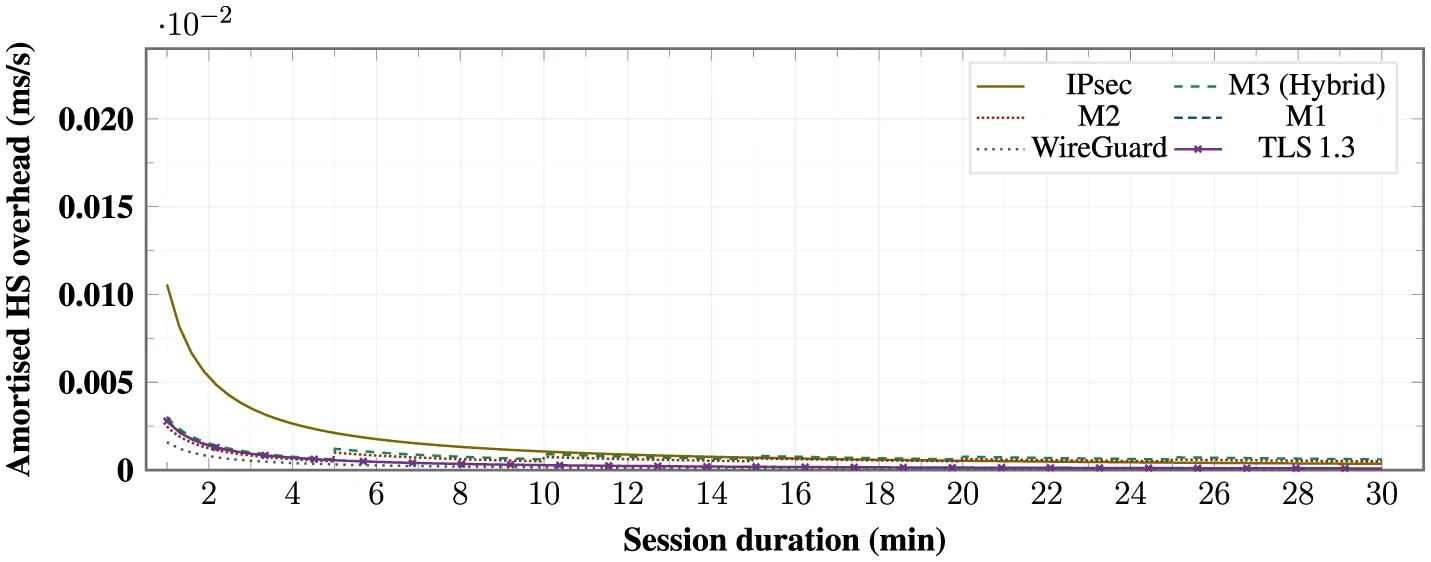
Amortized handshake overhead (ms per session-second) as a function of session duration, computed from measured handshake-processing latency and the configured refresh schedule. M2 and M3 include a periodic forward-secrecy refresh every 300 s. The overhead decays rapidly with session length and becomes negligible for typical BCI sessions. The plotted curves use the directly measured ARM64 protocol-processing times; the separate Cortex-M4 wearable projection is reported in Table 11.

Figure 11 shows the amortized session overhead computed from the measured ARM64 handshake-processing costs and the configured 300 s public-key refresh interval for M2 and M3. The per-second processing overhead decreases rapidly as session duration increases because setup cost is spread over a longer interval. M2 and M3 show small step increases at refresh points, while IPsec has the highest processing overhead for short sessions in the reported baseline. The complementary Cortex-M4 projection in Table 11 quantifies the longer setup time, peak stack, and mW-scale processing energy expected on a wearable-class MCU.

**Table 11:** Cycle-derived Cortex-M4 execution-time, peak-stack, and CPU-processing-energy projection for the PQ-NeuroLink post-quantum primitives.

Operation	Cycles (mean)	Time at 64 MHz (ms)	Peak stack (KiB)	Energy at 10 mW (mJ)	Energy at 20 mW (mJ)
ML-KEM-768 key generation	642,096	10.03	5.27	0.100	0.201
ML-KEM-768 encapsulation	658,754	10.29	6.32	0.103	0.206
ML-KEM-768 decapsulation	707,827	11.06	6.30	0.111	0.221
ML-DSA-44 key generation	1,426,025	22.28	37.40	0.223	0.446
ML-DSA-44 signing (mean)	3,943,121	61.61	43.77	0.616	1.232
ML-DSA-44 verification	1,421,623	22.21	8.70	0.222	0.444

Cycle counts and peak-stack values are from the PQM4 benchmark table for the speed-oriented ML-KEM-768 and ML-DSA-44 implementations (Benchmarks, 2026). Execution time is *C*_op_/64 MHz, and the energy columns include CPU processing only. ML-DSA signing is probabilistic: PQM4 reports approximately 1.81–17.01 million cycles around the 3.94-million-cycle mean. The stack-oriented ML-KEM-768 implementation reduces peak stack to approximately 2.8 KiB with a small cycle increase; the stack-oriented ML-DSA-44 implementation reduces signing stack to approximately 5.0 KiB while increasing mean signing time to approximately 189.6 ms at 64 MHz.

**Figure 12:**
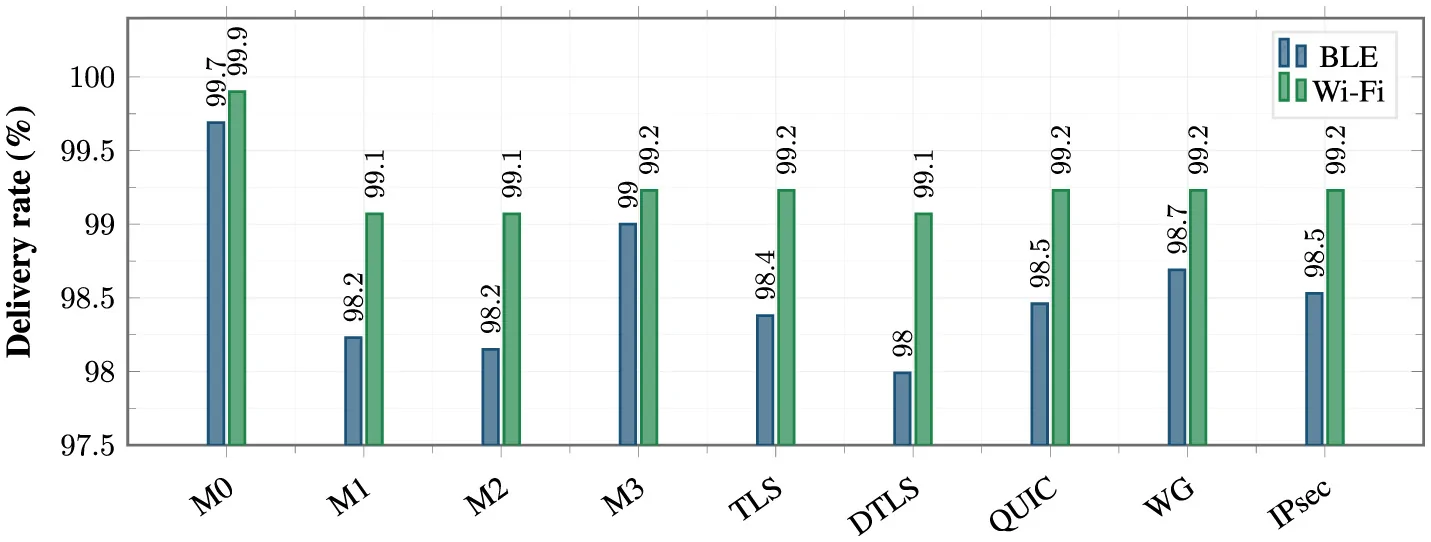
Measured packet delivery rate (%) under BLE (hatched) and Wi-Fi (solid) for all nine protocols. Delivery remains high across all configurations, with the dominant losses determined by link conditions rather than by the cryptographic protocol itself. M3 remains fully competitive with the classical alternatives on both links.

Figure 12 reports the measured packet delivery rate under the BLE-like and Wi-Fi-like links. Delivery remains high across all protocols, with BLE-like conditions slightly lower because of harsher link assumptions. M3 achieves 99.0% delivery on BLE and 99.23% on Wi-Fi, placing it within the top-performing group. Table 9 summarizes the most constrained BLE 1-hop setting: relative to M0, moving to M3 increases *p*_95_ by 0.45 ms while adding ML-KEM category-3 post-quantum key establishment, with delivery reduced by 0.69 percentage points.

#### 4.7.1 MCU-informed CPU, memory, and energy projection

Table 11 complements the measured ARM64 results with a wearable-class Cortex-M4 projection derived from the current PQM4 speed-oriented cycle and peak-stack measurements. The projection uses a 64 MHz clock and two active-CPU profiles, 10 mW and 20 mW, representing efficient and conservative microcontroller operating points grounded in the device specifications discussed in Section 3.3.4. These values are cycle-derived projections and are kept separate from the measured ARM64 timings in Figure 2.

The Cortex-M4 projection changes the interpretation of setup cost without changing the steady-state design conclusion. ML-KEM-768 operations require approximately 10–11 ms and 0.10–0.22 mJ over the 10–20 mW sensitivity range. ML-DSA-44 signing and verification project to approximately 61.6 ms and 22.2 ms, corresponding to 0.62–1.23 mJ and 0.22–0.44 mJ, respectively. These costs occur during authenticated setup or infrequent public-key refresh rather than for every EEG frame. The main embedded constraints are therefore transient setup latency, peak working memory, and reconnect energy; the continuous path remains dominated by symmetric AEAD, packetization, and the wireless link.

A complete M3 handshake projection is role dependent because the headset and hub execute different combinations of key generation, encapsulation, decapsulation, signing, and verification. Operation-level reporting therefore avoids assigning a single unsupported wearable handshake total and allows a deployment to compose the relevant costs for its selected endpoint roles and implementation profile.

The results in Table 9 confirm the descriptive trend in the most constrained summarized condition: M3 provides the strongest evaluated hybrid security configuration while preserving near-baseline steady-state latency in the BLE 1-hop model. Relative to M0, upgrading to M3 increases BLE 1-hop *p*_95_ latency by 0.45 ms, while delivery decreases by 0.69 percentage points and ML-KEM category-3 post-quantum key establishment is added. The practical interpretation is that the dominant latency contributors remain link, topology, and packetization effects rather than the choice among secured modes sharing the same symmetric streaming fast path.

## 5 Discussion

The results support the central design principle that latency-sensitive BCI telemetry should keep asymmetric cryptography off the per-frame path. In PQ-NeuroLink, post-quantum operations are restricted to session establishment and public-key refresh, while continuous EEG frames and control messages use lightweight authenticated encryption, counters, epochs, and replay checks. This separation is particularly relevant for neuroengineering scenarios in which degraded timing or feedback instability can affect user control, learning, or comfort. The results also clarify the source of post-quantum overhead: in the evaluated configuration, the dominant PQC cost is handshake transcript size rather than steady-state packet processing.

The comparison with TLS 1.3, QUIC, WireGuard, and IPsec should be interpreted as a secure-channel profiling study rather than as a claim that mature general-purpose protocols are unsuitable for BCI systems. PQ-NeuroLink contributes BCI-specific binding between the secure-channel state and the control-loop requirements: the session identifier, epoch, message type, sequence number, timestamp policy, replay window, key-update cadence, and safe-mode response are treated as part of the timing and safety model. This provides a narrower but more application-aware profile than general transport benchmarking alone.

### 5.1 Clinical, regulatory, and patient-safety implications

For clinical or home rehabilitation use, PQ-NeuroLink should be integrated into a broader safety and regulatory framework. Patient safety requires that authentication failure, replay detection, stale feedback, or severe latency anomaly transition the application to a predefined safe state. For robotic or prosthetic control this may mean stop or hold-last under a watchdog; for stimulation-bearing systems it should mean stimulation disablement until the channel is re-established and policy checks pass. Regulatory deployment also requires cybersecurity risk management, secure update procedures, audit logs, incident response, privacy controls for stored EEG and derived features, usability evaluation, and documentation of residual risk.

The timing watchdog is intentionally hysteretic. Cryptographic integrity, replay, and session-state violations remain immediate safe-state triggers, whereas timing-only anomalies require three consecutive missed or stale critical updates in the default profile. This policy avoids treating one ordinary wireless loss as a security emergency while bounding continued operation under persistent delay or jamming to 60–120 ms for the evaluated update cadence, plus the local shutdown time. The three-update threshold is a reference communication policy rather than a universal clinical constant: stimulation-bearing systems may select a stricter value, while non-actuating EEG monitoring may tolerate a larger value after application-specific control-stability and safety analysis.

### 5.2 Scope of interpretation and deployment path

The present results should be interpreted as communication-layer evidence obtained from EEG-derived replay and controlled link profiles. Hardware-specific effects such as radio firmware behavior, CPU governors, memory pressure, thermal throttling, battery-management policy, user mobility, and stimulation-device timing are platform-dependent and should be instantiated during device integration. Similarly, the active security tests exercise injection, replay, delay, MITM/session-splicing, and downgrade behavior at the communication layer, while compromised-hub, compromised-cloud, supply-chain, and large-scale revocation risks are handled through architectural controls. This scope provides a clear deployment path: implement the same profile on target headset and hub hardware, record CPU utilization, peak memory, power, and mobility behavior, and reuse the paired statistical workflow to confirm timing and security margins for the intended BCI task.

## 6 Conclusion

This paper presented PQ-NeuroLink, a latency-aware secure communication framework for distributed EEG-based BCI pipelines that targets active communication threats and migration toward post-quantum-ready session establishment. The core design choice is to keep post-quantum and hybrid public-key operations off the per-frame streaming path, using them primarily for session setup, endpoint identity binding, and infrequent public-key refresh, while protecting continuous telemetry with symmetric AEAD, sequence numbers, epochs, and replay defenses.

In the prototype evaluation using EEG-derived traffic replay over BLE-like and Wi-Fi-like links, with 1-hop and 2-hop topologies and injected loss, reordering, and tampering, post-quantum and hybrid modes increase session setup bytes but have limited effect on steady-state streaming latency under the tested conditions. The resource analysis now combines the measured ARM64 results with PQM4 cycle/stack data and representative 10–20 mW Cortex-M4 active-CPU profiles. The safety policy also distinguishes immediate cryptographic-failure handling from a three-update timing watchdog that bounds the communication contribution to time-to-safe-state.

Overall, PQ-NeuroLink shows that post-quantum-ready authenticated session establishment can be incorporated into distributed EEG communication prototypes without large steady-state latency penalties when public-key work is excluded from the per-frame path. The framework provides a reproducible basis for subsequent embedded, mobile, multi-user, and human-in-the-loop BCI studies that require both low-latency operation and long-horizon post-quantum security.

## Data Availability

The original contributions presented in the study are included in the article/supplementary material, further inquiries can be directed to the corresponding author.
